# Supplementary material for: Underwater paleontology inside cenotes reveals the Miocene-Pliocene fish diversity in the Yucatan Peninsula, southeast Mexico
Source: PLoS One. 2025 Feb 6;20(2):e0315382. doi: 10.1371/journal.pone.0315382 (PMC11801553; doi:10.1371/journal.pone.0315382)
Supplement: S1 Table — The asterisk symbol (*) indicates uncertainty, and the interrogation symbol (?) indicates that the measurement was impossible to determine due to specimen preservation. The symbol N/A means that this feature is not applicable in this specimen. (DOCX) [file pone.0315382.s003.docx]

**S1 Table. Measures and proportions related to the total height in the elasmobranch specimens studied.**

|  | **Total height (TH)** | **Total length** | **Crown height** | **Root height** | **Mesial cutting-edge angle** | **Root angle** |
| --- | --- | --- | --- | --- | --- | --- |
| **IGM 13913** | 11.18 | 15.22 | 6.81 (60.9% TH) | 3.98 (35.6% TH) | 38.17º | 15.46º |
| **IGM 13914** | 6.12 | 6.75* | 5 (81.7% TH*) | 2.91* (47.5% TH*) | 42.5º | 18.97º* |
| **IGM 13915** | 10.38 | 15.03 | 7.88 (75.9% TH) | 4.88 (47% TH) | 43.03º | 18.84º |
| **IGM 13916** | 8.62 | 10.86* | 6.26 (72.6% TH) | 3.84 (44.5% TH) | 40.6º | 13.63º |
| **IGM 13917** | 18.31 | 17.48 | 14.11 (77.1% TH) | 10.83 (59.1% TH) | 35.02º | 24.71º |
| **IGM 13918** | 15.43 | 15.49 | 12.21 (79.1% TH) | 9.45 (61.2% TH) | 35.11º | 22.9º |
| **IGM 13919** | 12.22* | 12.11* | 9.62* (78.7% TH*) | 5.78* (47.3% TH*) | 34.1º* | ? |
| **IGM 13920** | 13.39* | 15.53* | 9.32* (69.6% TH*) | 6.21* (46.3% TH*) | 37.3º* | ? |
| **IGM 13921** | 5.14 | 7.07 | 3.63 (70.6% TH) | 2.53 (49.2% TH) | 34.05º | 10.65º |
| **IGM 13922** | 12.83 | 18.27 | 9.82 (76.5% TH) | 6.64 (51.75% TH) | 37.25º | 10.2º |
| **IGM 13923** | 7.81 | 10.45 | 6.01 (76.9% TH) | 3.36 (43% TH) | 39.65º | 14.57º |
| **IGM 13924** | 24.86 | 17.19* | 18.57 (74.7% TH) | 13.14 (52.8% TH) | 27.44º | 30.18º |
| **IGM 13925** | 10.76* | 9.89* | 8.57* (79.6% TH) | 5.13 (47.7% TH*) | 29.15º | ? |
| **IGM 13926** | 46.68 | 37.51 | 32.13 (68.8% TH) | 26.74 (57.5% TH) | 27.83º | 26.05º |
| **IGM 13927** | 59.93* | 27.96* | 47.14 (78.6% TH*) | 26.87* (44.8% TH*) | 20.72º | ? |
| **IGM 13928** | 39.54* | 39.03* | 39.14 (98.9% TH*) | ? | 22.22º | ? |
| **IGM 13929** | 29.21 | 26.98* | 21.7 (74.3% TH) | 15.81 (54.1% TH) | 27.79º | 23.18º |
| **IGM 13930** | 28.36* | 18.12* | 20.8 (73.3% TH*) | 8.76* (30.9% TH*) | 22.69º | ? |
| **IGM 13931** | 4.16* | 26.92 | ? | 2.4 | N/A | N/A |
| **IGM 13932** | 3.6* | 15.79* | ? | 2.33 | N/A | N/A |
| **IGM 13933** | 1.9* | 12.86 | ? | 1.14 | N/A | N/A |

Measures are in millimeters. The asterisk symbol (*) indicates uncertainty, and the interrogation symbol (?) indicates that the measurement was impossible to determine due to specimen preservation. The symbol N/A means that this feature is not applicable in this specimen.
