# Supplementary material for: Underwater paleontology inside cenotes reveals the Miocene-Pliocene fish diversity in the Yucatan Peninsula, southeast Mexico
Source: PLoS One. 2025 Feb 6;20(2):e0315382. doi: 10.1371/journal.pone.0315382 (PMC11801553; doi:10.1371/journal.pone.0315382)
Supplement: Attachment — Submitted filename: PONE-D-24-07932_REVIEWED.pdf [file pone.0315382.s004.pdf]

PLOS ONE

Underwater paleontology inside cenotes reveals the Miocene-Pliocene fish diversity in the Yucatan Peninsula, southeast Mexico

--Manuscript Draft--

**Manuscript Number:**	PONE-D-24-07932
**Article Type:**	Research Article
**Full Title:**	Underwater paleontology inside cenotes reveals the Miocene-Pliocene fish diversity in the Yucatan Peninsula, southeast Mexico
**Short Title:**	Miocene-Pliocene fishes from the Carrillo Puerto Formation
**Corresponding Author:**	Kleyton Cantalice, Ph.D.<br>Universidad Nacional Autónoma de México: Instituto de Geología<br>Ciudad de México, Ciudad de México MEXICO
**Keywords:**	Cenotes; Fossils; Elasmobranch; Diodontidae; Miocene-Pliocene; Carrillo Puerto Formation; Yucatan Peninsula, Mexico
**Abstract:**	The Yucatan Peninsula, southeast México, is home to one of the largest karstic aquifers with the longest underwater cave in the world and a complex system of sinkholes, locally called cenotes. This system is developed on a great Late Miocene to Early Pliocene carbonate platform belonging to the Carrillo Puerto Formation. The karstification process partially erodes the surface of this deposit and gradually reveals the fossil diversity on the strata of the Carrillo Puerto Formation. Here we present the fossil fish diversity found in underwater prospections in the Cenotes Sambulá, San Juan, and X-Nabuy. Our results indicate the presence of at least 11 different taxa, which include: 1) species that live today on Mexican coasts, such as Carcharhinus brachyurus, C. leucas, C. perezii, Carcharodon carcharias, and unidentified Rhinoptera species; 2) species completely extinguished, such as †Galeocerdo mayumbensis, †Hemipristis serra, and †Otodus (Carcharocles) megalodon; 3) species that are not currently distributed in Mexican coasts Carcharhinus macloti, and Pteromylaeus species. Furthermore, a new Diodontidae species †Chilomycterus dzonotensis sp. nov. is described. It represents an increment in the Neogene fish diversity in the Gulf of Mexico and corroborates a shallow marine environment associated with a coral reef system. Furthermore, the small size of all teeth indicates that the deposits of Carrillo Puerto were a shelter for small marine organisms, and the presence of some taxa highlights local extinctions in the Western Atlantic during the Late Cenozoic.
**Order of Authors:**	Kleyton M. Cantalice, Ph.D.
	Hugo E. Salgado-Garrido
	Erick Sosa-Rodríguez
	Kay Vilchis-Zapata
	Gerardo González-Barba, PhD.
**Additional Information:**	
**Question**	**Response**
**Financial Disclosure**<br><br>Enter a financial disclosure statement that describes the sources of funding for the work included in this submission. Review the submission guidelines for detailed requirements. View published research articles from *PLOS ONE* for specific examples.	Yes

This statement is required for submission and **will appear in the published article** if the submission is accepted. Please make sure it is accurate.<br><br>**Funded studies**<br>Enter a statement with the following details:<br>• Initials of the authors who received each award<br>• Grant numbers awarded to each author<br>• The full name of each funder<br>• URL of each funder website<br>• Did the sponsors or funders play any role in the study design, data collection and analysis, decision to publish, or preparation of the manuscript?<br><br>Did you receive funding for this work?	
Please add funding details.<br>as follow-up to "**Financial Disclosure**<br><br>Enter a financial disclosure statement that describes the sources of funding for the work included in this submission. Review the submission guidelines for detailed requirements. View published research articles from *PLOS ONE* for specific examples.<br><br>This statement is required for submission and **will appear in the published article** if the submission is accepted. Please make sure it is accurate.<br><br>**Funded studies**<br>Enter a statement with the following details:<br>• Initials of the authors who received each award<br>• Grant numbers awarded to each author<br>• The full name of each funder<br>• URL of each funder website<br>• Did the sponsors or funders play any role in the study design, data collection and analysis, decision to publish, or preparation of the manuscript?	KMC. Grant Number: IA206123. Dirección Genral de Asuntos del Personal Académico (DGAPA) - Programa de Apoyo a Proyectos de Investigación e Innovación Tecnológica (PAPIIT).

Did you receive funding for this work?"	
Please select the country of your main research funder (please select carefully as in some cases this is used in fee calculation).<br>as follow-up to "**Financial Disclosure**<br><br>Enter a financial disclosure statement that describes the sources of funding for the work included in this submission. Review the submission guidelines for detailed requirements. View published research articles from *PLOS ONE* for specific examples.<br><br>This statement is required for submission and **will appear in the published article** if the submission is accepted. Please make sure it is accurate.<br><br>**Funded studies**<br>Enter a statement with the following details:<br>• Initials of the authors who received each award<br>• Grant numbers awarded to each author<br>• The full name of each funder<br>• URL of each funder website<br>• Did the sponsors or funders play any role in the study design, data collection and analysis, decision to publish, or preparation of the manuscript?<br><br>Did you receive funding for this work?"	MEXICO - MX
**Competing Interests**<br><br>Use the instructions below to enter a competing interest statement for this submission. On behalf of all authors, disclose any competing interests that could be perceived to bias this work—acknowledging all financial support and any other relevant financial or non-financial competing interests.<br><br>This statement is required for submission and **will appear in the published article** if	The authors have declared that no competing interests exist

the submission is accepted. Please make sure it is accurate and that any funding sources listed in your Funding Information later in the submission form are also declared in your Financial Disclosure statement.<br><br>View published research articles from *PLOS ONE* for specific examples.<br><br>**NO authors have competing interests**<br><br>Enter: *The authors have declared that no competing interests exist.*<br><br>**Authors with competing interests**<br><br>Enter competing interest details beginning with this statement:<br><br>*I have read the journal's policy and the authors of this manuscript have the following competing interests: [insert competing interests here]*<br><br>* typeset	
**Ethics Statement**<br><br>Enter an ethics statement for this submission. This statement is required if the study involved:<br><br>• Human participants<br>• Human specimens or tissue<br>• Vertebrate animals or cephalopods<br>• Vertebrate embryos or tissues<br>• Field research<br><br>Write "N/A" if the submission does not require an ethics statement.<br><br>General guidance is provided below. Consult the submission guidelines for detailed instructions. **Make sure that all information entered here is included in the Methods section of the manuscript.**	N/A

**Format for specific study types**<br><br>**Human Subject Research (involving human participants and/or tissue)**<br>• Give the name of the institutional review board or ethics committee that approved the study<br>• Include the approval number and/or a statement indicating approval of this research<br>• Indicate the form of consent obtained (written/oral) or the reason that consent was not obtained (e.g. the data were analyzed anonymously)<br><br>**Animal Research (involving vertebrate animals, embryos or tissues)**<br>• Provide the name of the Institutional Animal Care and Use Committee (IACUC) or other relevant ethics board that reviewed the study protocol, and indicate whether they approved this research or granted a formal waiver of ethical approval<br>• Include an approval number if one was obtained<br>• If the study involved *non-human primates*, add *additional details* about animal welfare and steps taken to ameliorate suffering<br>• If anesthesia, euthanasia, or any kind of animal sacrifice is part of the study, include briefly which substances and/or methods were applied<br><br>**Field Research**<br><br>Include the following details if this study involves the collection of plant, animal, or other materials from a natural setting:<br>• Field permit number<br>• Name of the institution or relevant body that granted permission	
**Data Availability**<br><br>Authors are required to make all data underlying the findings described fully available, without restriction, and from the time of publication. PLOS allows rare exceptions to address legal and ethical concerns. See the PLOS Data Policy and FAQ for detailed information.	Yes - all data are fully available without restriction

A Data Availability Statement describing where the data can be found is required at submission. Your answers to this question constitute the Data Availability Statement and **will be published in the article**, if accepted.<br><br>**Important:** Stating 'data available on request from the author' is not sufficient. If your data are only available upon request, select 'No' for the first question and explain your exceptional situation in the text box.<br><br>Do the authors confirm that all data underlying the findings described in their manuscript are fully available without restriction?	
**Describe where the data may be found in full sentences. If you are copying our sample text, replace any instances of XXX with the appropriate details.**<br><br>• If the data are **held or will be held in a public repository**, include URLs, accession numbers or DOIs. If this information will only be available after acceptance, indicate this by ticking the box below. For example: *All XXX files are available from the XXX database (accession number(s) XXX, XXX.).*<br>• If the data are all contained **within the manuscript and/or Supporting Information files**, enter the following: *All relevant data are within the manuscript and its Supporting Information files.*<br>• If neither of these applies but you are able to provide **details of access elsewhere**, with or without limitations, please do so. For example:<br><br>*Data cannot be shared publicly because of [XXX]. Data are available from the XXX Institutional Data Access / Ethics Committee (contact via XXX) for researchers who meet the criteria for access to confidential data.*<br><br>*The data underlying the results presented in the study are available from (include the name of the third party*	All relevant data are within the manuscript and its Supporting Information files.

and contact information or URL).<br>• This text is appropriate if the data are owned by a third party and authors do not have permission to share the data.<br><br>* typeset	
Additional data availability information:	Tick here if the URLs/accession numbers/DOIs will be available only after acceptance of the manuscript for publication so that we can ensure their inclusion before publication.

Powered by Editorial Manager® and ProduXion Manager® from Aries Systems Corporation

Manuscript

Click here to access/download;Manuscript;Cantalice_et_al_2024_Plos_Main_

Underwater paleontology inside cenotes reveals the Miocene-Pliocene fish diversity in the Yucatan Peninsula, southeast Mexico

Kleyton M. Cantalice[1]*, Hugo E. Salgado-Garrido[2¶], Erick Sosa-Rodríguez[3&], Kay Vilchis-Zapata[4&], Gerardo González-Barba[5¶], on behalf of the Underwater Archaeological Atlas project^

[1]Departamento de Paleontología, Instituto de Geología, Universidad Nacional Autónoma de México, Ciudad de México, México.

[2]Laboratorio de Carbonatos y Procesos Kársticos, Instituto de Geología, Universidad Nacional Autónoma de México, Ciudad de México, México.

[3]Exploración Subterránea Responsable, Mérida, Yucatán, México.

[4]Secretaria de Desarrollo Sustentable del Estado de Yucatán, Mérida, Yucatán, México.

[5]Departamento Académico de Ciencias Marinas y Costeras, Universidad Autónoma de Baja California Sur, La Paz, Baja California Sur, México.

*Corresponding author

E-mail: kleytonmc@geologia.unam.mx (KMC)

¶ These authors contributed equally to this work.
& These authors also contributed equally to this work.

^Membership of the Underwater Archaeological Atlas project is provided in the Acknowledgments

Short title: Miocene-Pliocene fishes from the Carrillo Puerto Formation

Abstract

The Yucatan Peninsula, southeast México, is home to one of the largest karstic aquifers with the longest underwater cave in the world and a complex system of sinkholes, locally called cenotes. This system is developed on a great Late Miocene to Early Pliocene carbonate platform belonging to the Carrillo Puerto Formation. The karstification process partially erodes the surface of this deposit and gradually reveals the fossil diversity on the strata of the Carrillo Puerto Formation. Here we present the fossil fish diversity found in underwater prospections in the Cenotes Sambulá, San Juan, and X-Nabuy. Our results indicate the presence of at least 11 different taxa, which include: 1) species that live today on Mexican coasts, such as *Carcharhinus brachyurus*, *C. leucas*, *C. perezii*, *Carcharodon carcharias*, and unidentified *Rhinoptera* species; 2) species completely extinguished, such as †*Galeocerdo mayumbensis*, †*Hemipristis serra*, and †*Otodus* (*Carcharocles*) *megalodon*; 3) species that are not currently distributed in Mexican coasts *Carcharhinus macloti*, and *Pteromylaeus* species. Furthermore, a new Diodontidae species †*Chilomycterus dzonotensis* sp. nov. is described. It represents an increment in the Neogene fish diversity in the Gulf of Mexico and corroborates a shallow marine environment associated with a coral reef system. Furthermore, the small size of all teeth indicates that the deposits of Carrillo Puerto were a shelter for small marine organisms, and the presence of some taxa highlights local extinctions in the Western Atlantic during the Late Cenozoic.

Introduction

Cenote derives from the Maya word *ts'onot* or *d'zonot* to describe steep-walled natural wells generally opened by a cave roof collapse (collapse doline) or solution sinkhole. This structure provides access to the water table and extends below it through a complex system of underground galleries [1]. In the Yucatan Peninsula, intense karstification processes have created one of the largest karst aquifers with many underwater and dry caves, including the world's longest underwater cave the Sac Actun System in Quintana Roo state [2], and more than 3000 cenotes recorded in the state of Yucatan [3].

The freshwater that fills the cenotes is extremely important, since it is currently the principal water supply for human activities in the region, and its distinctive environment provides a habitat for endemic organisms [4,5]. Despite their relevance, these groundwater systems are fragile and highly vulnerable to pollution and overexploitation due to the pressure of cities growing [6].

The cenotes are objects of interest for many branches of scientific investigations including biology [4], hydrogeology [5,7], cartography [8], and archeology [9,10]. Earth science research indicates that the complex underwater caves and cenote system of the Yucatan Peninsula are mainly developed in sedimentary rocks of the Carrillo Puerto Formation, which ranges in age from Late Miocene to Early Pliocene [11].

A great portion of the Carrillo Puerto Formation is formed by coquina strata, which evidence the presence of several fossil invertebrates, such as bivalves, gastropods, and corals, among other marine organisms [12–14]. Despite the abundance of ancient marine

biodiversity on the sediments that compose the cenotes, the paleontological understanding of this geological formation is still incipient since the fossil record remains almost completely unidentified. This study aims to present the fish diversity from the Carrillo Puerto Formation obtained through underwater prospections in three distinct cenotes along the Yucatan State, southeast Mexico.

Geological settings

The Carrillo Puerto Formation corresponds to the most outcropping limestone on the Yucatan Peninsula, covering an area of about 8800 km^2, which represents the central and eastern part of the state of Quintana Roo, the central region of Yucatán, and northeastern Campeche (Fig 1). It comprises a carbonate package of mudstone, wackestone, grainstone, packstone, boundstone, and calcareous breccias, with thicknesses ranging from five to 25 meters [14].

Fig 1. Location map of fossil collection.

(A) Geological map with formations Icaiche, Chichén Itza, Carrillo Puerto, and Quaternary deposits (Q) modified from SGM (2006) [15]. Paleontological records. 1 (Cenote Kambul) and 2 (Rancho Las Chapas) correspond to Sirenid for Domning [16,17]; while 3 Sambulá (Motul), 4 X-Nabuy (Suma), and 5 San Juan (Homún) cenotes correspond to this work. (B) Idealized stratigraphic unit of the Carrillo Puerto Formation. The cenote entrance is located at a different altimetric position meters above sea level (masl). Also, the water table (WT)

starts at a different position. Diving starts at WT. Each cenote has a maximum depth where the samples were collected, it corresponds to the maximum stratigraphic range (MSR) which includes the dive and meters above WT.

The Carrillo Puerto Formation is divided into four distinct sections: 1) the base, formed by thick coquina strata covered with solid, white, or yellowish limestone, varying from cream-colored bioclastic packstone and calcarenites [12]; 2) the middle-level, conformed by impure limestones and yellowish and reddish clays, which by alteration give rise to red lateritic clays; 3) the upper level, represented by white, hard and massive limestone [13]; 4) the karst dissolutions and erosions of the lapiaz type observed in several representative outcrops of the unit [18,19].

The Late Miocene to Early Pliocene age is attributed to the Carrillo Puerto Formation based on the presence of fossil foraminifera of the genera *Archaias* de Montfort, 1808, *Sorites* Ehrenberg, 1839, *Peneroplis* de Montfort, 1808 (*P. proteus* d'Orbigny, 1839), *Cycioloculina* Heron-Allen and Earland 1908, *Nonion* de Montfort, 1808, and *Gypsina* Carter, 1877; bivalves of the genera *Arca* Linnaeus, 1758 and *Venus* Linnaeus, 1758; turritellas, ostracods, and Hexacorallia corals [12,18–20]. The Carrillo Puerto Formation correlates with La Laja, Depósito, and Encantado de la Cuenca Formations, located at Veracruz State, and overlies a small portion of the unidentified Oligocene formation and the great portion of the Eocene Chichén Itzá Formation [18] (Fig 1).

Based on the lithological and faunal characteristics, it is considered a shallow shelf depositional environment with internal neritic conditions and middle neritic influence. These

conditions correspond to a reasonably long period, which must have covered a large part of the Peninsula where abundant fossils were developed in low-energy areas [14,18]. The Elasmobranch and the teleost fossil remain herein described from the Carrillo Puerto Formation represent an increment in the knowledge about the marine diversity on the Gulf of Mexico during the Neogene and highlight local extinctions on the Western Atlantic after the Miocene-Pliocene boundary.

Material and methods

A total of 22 fossil specimens were studied. Elasmobranchs and a teleost were collected in three cenotes (Sambulá, X-Nabuy, and San Juan) located in Yucatan state during the field trip in April 2023 (Fig 1A). The underwater collection used cave-diving vertical progression techniques and equipment, with Nitrox 32% tanks and side-mount configuration (Fig 2 A-B, S1 File). During the diving, the depth measures were taken using a dive computer Teric Shearwater (Fig 2A). Dives for collections were conducted by two specialized divers for 60 minutes for each person. The maximum depth reached was 25 meters in the Cenote San Juan (Fig 1B). In one of the cenotes, cave decompression techniques with multi-stations and rebreathers were also used, given the depth and bottom time. The specimens were removed from the rock using a chisel and hammer (Fig 2B-F, S2 File). The samples were deposited in plastic bags to be transported to the surface and safeguarded in cotton wool.

Fig 2. Dive and sampling techniques inside cenotes.

(A) Side-mount diving technique. (B-E) Sample technique using chisel and hammer. (F) Tooth embedded in the wall.

The fossil material was cleaned using a micro jacket 2 tool and needles under a stereomicroscopic observation. In some cases, 0.5% sulfamic acid solution was directly applied to help the removal of well-consolidated sediments. Photographs were taken using a Nikon D5500 DSLR camera with an AF-S Micro-Nikkor 60mm f/2.8G ED macro lens, and the figures were edited with the software Gimp v.2.10.34 and Inkscape v.1.2. The specimens are housed at the *Colección Nacional de Paleontología* [National Collection of Paleontology] of the *Universidad Nacional Autónoma de México* [Autonomous National University of Mexico] under the acronym IGM. Elasmobranch anatomic interpretations follow that of Purdy et al. [21], and porcupinefish osteology follows that of Tyler [22] and Aguilera et al. [23].

Abbreviations

IGM, *Instituto Geológico de México* [Mexican Institute of Geology], catalog registration abbreviation for specimen deposited in the *Colección Nacional de Paleontología* [National Collection of Paleontology of Mexico]. **IGM-loc.**, catalog abbreviation for fossil localities on the National Collection of Paleontology of Mexico. **INAH**, *Instituto Nacional de Antropología e Historia* [National Institute of Anthropology and History]. **UNAM**, *Universidad Nacional Autónoma de México* [Autonomous National University of Mexico].

Results

Systematic Paleontology

Filo Chordata

Infraclass Elasmobranchii Bonaparte, 1838 [24]

Division Selachii Cope, 1871 [25]

Order Carcharhiniformes Compagno, 1973 [26]

Family Carcharhinidae Jordan and Evermann, 1886 [27]

Genus *Carcharhinus* Blainville, 1816 [28]

Carcharhinus brachyurus Günther, 1870 [29]

(Fig 3)

Fig 3. *Carcharhinus brachyurus* Günther, 1870 [29] specimens (IGM 12301 to IGM 12303, and IGM 12318) from the Carrillo Puerto Formation.

(A, E, I, and M) Labial view. (B, F, J, and N) Lingual view. (C, G, K, and O) Right lateral view. (D, H, L, and P) Left lateral view. The scale bars indicate 3 mm.

Referred material

IGM 12301; a complete tooth with the crown turned to the right. IGM 12302; a small tooth with the crown turned to the right and the root eroded. IGM 12303; a complete tooth

with the crown turned to the left. IGM 12318; a tooth with the crown turned to the left and a small part of the distal root lobe absent. All the specimens were collected in the cenote X-Nabuy.

Description

There are four teeth ranging from 6.9 to 13.6 millimeters (mm) in total length and from 6.5 to 8.7 mm in depth. The teeth are flattened labio-lingually and have a narrow triangular crown. Lingually, the crown represents about 76% of the maximum depth, and the root about 46% of the maximum depth. The crown is triangular and narrow, with the mesial and distal cutting edges slightly curved and serrated. In the lateral view, the labial apex projection and the sigmoid curvature are pronounced in IGM 12301 and IGM 12318 (Fig 3). A deep and acute notch separates the crown and shoulder on the distal surface. The shoulders are laterally large and enameled. The serrations are vestigial in the apical portion of the crown, becoming more developed over the shoulders, and coarse on the distal cutting edge in IGM 12302 and IGM 12303. Secondary serrations and cusplet are lacking. The ventral edge curvature of the crown is not deep; the distance between the middle point of the crown's ventral edge through the ventral tip of the mesial cutting edge forms an angle of about 21 degrees. The ventral edge of the root is also shallow; the distance between the ventral extreme of the mesial root lobe to the forms an angle of about 14 degrees. Medial protuberance is well-developed. The central foramen is in a deep transverse groove. The transversal groove is notable in both labial and lingual views (Fig 3).

Remarks

The diagnostic features that indicate these specimens as *Carcharhinus brachyurus* (the Copper shark) include the narrow triangular, strongly serrated, and oblique crown. Furthermore, the curvature of the distal cutting edge forms a deep acute angle, separating the serration of the shoulders from the apex of the crown [21,30].

The species *Carcharhinus brachyurus* is distributed in other Miocene-Pliocene fossil localities from Brazil, Colombia, Venezuela, USA, Portugal, and France [21,31–33]. In Mexico, specimens of *C. brachyurus* are reported from the Miocene-Pliocene strata of Baja California [34–36]. Living specimens have a worldwide distribution, inhabiting coastal environments on both sides of Mexico. Extant *C. brachyurus* reaches up to 320 m in marine environments and is generally associated with inshore and reef systems; however, it is also reported in estuaries and occasionally in freshwater [37].

Carcharhinus leucas Valenciennes, 1839 [38]

(Fig 4)

Fig 4. *Carcharhinus leucas* Valenciennes, 1839 [38] specimens (IGM 12304 to IGM 12307) from the Carrillo Puerto Formation.

(A, E, I, and M) Labial view. (B, F, J, and N) Lingual view. (C, G, K, and O) Right lateral view. (D, H, L, and P) Left lateral view. The scale indicates 5 mm.

Referred material

IGM 12304; a complete tooth with the crown turned to the left. IGM 12305; an almost complete tooth with the crown turned to the left, the cutting edges are damaged, and a small portion of the medial lobe of the root is lost. IGM 12306; an eroded tooth with the crown turned to the left, the incomplete root, and the cutting edges are vestigial. IGM 12307; a very eroded crown turned to the right. All the specimens were collected in the cenote X-Nabuy.

Description

Four teeth ranging from 16.5 to 18.3 mm in length, and from 17.8 mm to 20.3 in depth in complete specimens. The teeth are labio-lingually flattened with a large triangular crown representing about 75% of the total depth, while the root represents about 60% of the total depth in lingual view. Both labial and lingual surfaces are straight; the lingual surface is inclined, and the apex is not labially curved (Fig 4). A medial constriction on the medial portion of the crown produces a curvature in both cutting edges, which is more pronounced on the distal edge. The distance between the apex and the base of the mesial cutting edge forms an angle of about 126 degrees, while from the apex to the distal cutting edge forms an angle of about 65 degrees. Both cutting edges are serrated. The serrations are fine close to the apex and become coarse in the base of the crown (Fig 4). Cusplet are lacking. The ventral edge of the crown is arcuate, the distance between the middle point of the ventral edge of the crown through the ventral tip of the mesial cutting edge forms an angle of about 40 degrees. The root presents a deep curvature on the ventral edge. The distance between the tip of the

mesial root lobe to the medial portion of the root forms an angle of about 24 degrees. The medial protuberance is not strongly pronounced. The central foramen is present, and the transverse groove is lacking (Fig 4).

Remarks

The tooth is attributed to *Carcharhinus leucas* (the Bull shark) due to the labio-lingually flattened teeth, with a broad triangular crown and an oblique apex; the cutting edges shallow notched on both sides, but more developed on the distal surface; serrations along both cutting edges and more developed on the crown base and the shoulders; root arcuate showing a central foramen; and the lack of transverse groove [21,39].

The fossil record of *Carcharhinus leucas* dates to the Miocene and is distributed in outcrops from Brazil, Egypt, India, Panama, Perú, Portugal, Italy, Angola, USA, among others e.g., [40–42]. The first Mexican *C. leucas* fossil record is from Miocene-Pliocene outcrops of Baja California [34,35]. This species currently inhabits marine shallow waters less than 30 m depth; however, occasionally migrates to freshwater and deeper waters close to 150 m depth [30].

Carcharhinus macloti Müller & Henle, 1839 [43]

(Fig 5 A-D)

Fig 5. *Carcharhinus macloti* Müller & Henle, 1839 [43] (IGM 12308), *C. perezii* Poey, 1876 [44] (IGM 12309), and *Carcharhinus* sp. (IGM 12310) from Carrillo Puerto Formation.

(A, E, and I) Labial view. (B, F, and J) Lingual view. (C, G, and K) Right lateral view. (D, H, and L) Left lateral view. The scale indicates 5 mm.

Referred material

IGM 12308; a complete tooth with the crown turned to the right. The specimen was collected in the cenote X-Nabuy.

Description

There is a unique tooth with 11.9 mm in total length and 10.5 mm in depth. The tooth is flattened labio-lingually, with the maximum depth of the crown representing about 70% of the depth of the tooth in the lingual view, and the root occupying 46% of the total depth in the same view. The crown is narrow and triangular, with the apex turned to the left in labial view (Fig 5C-D). The labial surface of the crown is straight; while the lingual is medially concave, forming the sigmoid curvature in the lateral view. The apex is slightly curved to the labial portion. The mesial cutting edge is straight and inclined, forming an angle of 148 degrees about the apex. The distal cutting edge is also straight and inclined, forming an angle of about 73 degrees with the apex. The distal cutting edge presents a deep and acute notch. Both mesial and distal cutting edges are smooth. Cusplets are lacking; however, the shoulders are large and enameled. The dorsal surface of the mesial shoulder is adorned with small and

weak serrations on the mesial side. On the distal shoulder, the serrations are more developed and present a small cusplet-like shape. The ventral edge of the crown is arcuate. The distance between the middle point of the ventral border of the crown through the ventral tip of the mesial cutting edge forms an angle of about 45 degrees. The root is also poorly arcuate and shallow. The distance between the middle point of the ventral border and the tip of the marginal root on the mesial lobe forms an angle of about 11 degrees. The lingual protuberance is triangular and pronounced. The base of the root lobes has a light cream color, and the external surface is ridged, mainly on the lingual view. Both the central foramen and transverse groove are well-developed. The transverse groove is visible on both lingual and labial views.

Remarks

The unique specimen herein studied is attributed to *Carcharhinus macloti* (the Hardnose shark) due to the small tooth with a narrow crown and smooth cutting edges in the apical portion of the crown and developed serrations on the shoulders, showing a cusplet-like shape on the distal surface [21,40]. *Carcharhinus macloti* is very similar to *Carcharhinus dilcemai* [45], from the Early Miocene of the East Pisco Basin, Chilcatay Formation, Peru [45]. The species herein described is more related to *C. macloti* due to the lack of the twist in the crow, a diagnostic feature of *C. dilcemai* [45].

The fossil record of *C. macloti* is scarce compared with other Requiem sharks. *Carcharhinus macloti* teeth were previously reported from the Early Miocene Caribbean seas of northeastern Amazonian, in Brazil; on Miocene Western North Atlantic marine outcrops from Maryland, and the Miocene-Pliocene from North Carolina [21,40,46]. The fossil herein

described is the first report from Miocene-Pliocene marine fossil beds from the Gulf of Mexico, indicating a wide distribution of *C. macloti* in the Western Atlantic coasts. Living Hardnose sharks are not distributed in the Atlantic but are restricted to continental and insular shelf environments from the Indo-West Pacific [40,47].

Carcharhinus perezii Poey, 1876 [44]

(Fig 5 E-H)

Referred material

IGM 12309; a well-preserved tooth with the crown turned to the left side. Only a small portion of the external border of the mesial root lobe is lacking. The specimen was collected in the cenote X-Nabuy.

Description

There is a unique labio-lingually flattened tooth with 17.2 mm of total length and 13.8 mm of total depth. The maximum depth of the crown represents about 74% of the depth of the tooth in the lingual view and the highest portion of the root represents about 43% of the depth. The enamel is well-conserved in the labial and lingual surfaces of the crown. The crown is triangular and narrow, with the apex directed to the right side on the labial view. The labial surface is almost straight, and the apex is not projected in lateral view. Both mesial and distal cutting edges are finely serrated in all the extensions (Fig 5 E-F); the serrations on

the apex are minute. The mesial cutting edge forms a curvature of 148 degrees, while the distal cutting edge is curved with the distal edge with a curvature of 67 degrees about the apex. The notch in the distal surface is not deep, and the cusplets are lacking. The shoulder is large and enameled, forming a blade that also is finely serrated. The root has a very pronounced lingual protuberance, with a well-defined central foramen embedded in a deep transverse groove visible in both labial and lingual views. The basal margin of the root is slightly curved; the line ranging from the middle of the root curvature to the mesial upper lobe comprises an angle of about 10 degrees.

Remarks

Although very similar to the teeth attributed to *Carcharhinus brachyurus*, this single specimen is attributed to *C. perezii* (the Caribbean reef shark) due to the presence of a large and triangular crown with vestigial serrations along both the cutting edges and the curvature of the cutting edges not pronounced [21,39,40]. The fossil record of *C. perezii* is commonly reported in Miocene-Pliocene outcrops from the USA, Panama, Portugal, Italy, and others [21,39,40,48]. It is the first fossil record of *C. perezii* from Mexico. Extant species are distributed in the western Atlantic, including the Gulf of Mexico and the Antilles [30,47]. *Carcharhinus perezii* is a reef-associated species, usually ranging from 1 to 35 meters [21,30].

Carcharhinus sp.

(Fig 5 I-L)

Referred material

IGM 12310; an almost complete tooth with only a little portion of the mesial root lobe lacking. The specimen was collected in the cenote San Juan.

Description

There is a unique tooth with 10.9 mm in total length and 8.5 mm in depth. The tooth is flattened labio-lingually and has a straight and triangular crown, with about 60% of the depth and the root occupying 46% of the total depth in lingual view. The tooth crown is triangular and narrow, with the apex pointing upwards. Both labial and lingual surfaces are curved, and the apex is labially curved, forming a lateral sigmoid curvature in lateral view. The mesial cutting edge is slightly convex (Fig 5 I-J), forming an angle of 124 degrees with the apex; while the distal cutting edge is straight and slightly inclined, and forms an angle of 37 degrees about the apex. Both mesial and distal cutting edges are smooth. The curvature of the medial portion of the ventral border of the crown is not very pronounced. The angle formed in a line from the ventral tip of the mesial cutting edge to the middle point of the ventral border of the crown represents 22 degrees. The shoulders are wide; however, the enameled portion over its dorsal surface is thin. There are no serrations or cusplets over the shoulders. The root is not deep; however, the lobes are large. The curvature in the ventral edge of the root is less pronounced. The distance between the middle point of the ventral border and the tip of the marginal root on the mesial lobe forms an angle of about 14 degrees. The medial protuberance in the lingual surface is almost triangular and very pronounced. The

central foramen is well-developed and embedded in a deep transversal groove visible in both labial and lingual views.

Remarks

The specimen is not attributed to any known species since only general features of *Carcharhinus* (Requiem sharks) are preserved. The features include the semi-erect crown with a smooth cutting edge, an enameled and large shoulder, and a large root with the central foramen and transverse groove notables. According to Purdy [39], these features are related to the lower jaw tooth of a general *Carcharhinus* species and, therefore, could belong to any of the species described above. For this reason, we prefer to maintain this specimen as a not-determined member of the genus *Carcharhinus*.

Genus *Galeocerdo* Müller & Henle 1837 [49]

†*Galeocerdo mayumbensis* Dartevelle & Casier, 1943 [50]

(Fig 6 A-D)

Fig 6. †*Galeocerdo mayumbensis* Dartevelle & Casier, 1943 [50] (IGM 12311; A-D) and †*Hemipristis serra* Agassiz, 1843 [51] (IGM 12312; E-F) from Carrillo Puerto Formation.

(A and E) Labial view. (B and F) Lingual view. (C and G) Right lateral view. (D and H) left lateral view. The scale bar from (A) to (D) represents 10 mm, while from (E) to (H) comprises 5 mm.

Referred material

IMG 12311; an incomplete tooth with the crown turned to the right, the lobe of the root, and the basal portion of the crown in the distal surface are lacking. The specimen was collected in the cenote Sambulá.

Description

There is a unique tooth with about 16.9 mm in total length and 25.3 mm in depth. The tooth is labio-lingually flattened. The maximum depth of the crown is about 77% of the teeth depth in the lingual view, and the highest portion of the root represents about 53% of the total depth at the same plan. The crown is triangular and large, with the apex directed to the right in labial view (Fig 6 A-B). The labial surface of the crown is concave, while the lingual is convex. An apex curvature is directed to the labial portion. The sigmoid curvature of the crown is absent (Fig 6 C-D). The mesial cutting edge is concave and forms an angle of 53 degrees to the apex. The distal cutting edge forms an angle of 113 degrees to the apex. The notch in the distal cutting edge is acute and shallow. The cutting-edge serration in the apical portion of the crown is thin; however, at the base of the crown, the serrations become coarse and triangular, showing secondary serrations (Fig 6 A-B). Both coarse and secondary serrations are more developed on the basal portion of the distal cutting edge. Cusplets are

lacking. The ventral border of the crown is arcuate; the distance between the middle point of the ventral edge of the crown through the ventral tip of the medial cutting edge forms an angle of about 37 degrees. The root is also deep and arcuate; the distance between the middle point of the ventral border of the root and the tip of the marginal root on the mesial root lobe forms an angle of about 34 degrees. The lingual protuberance is semicircular and less pronounced. The central foramen is present, and the transverse groove is lacking.

Remarks

The specimen here studied is attributed to the extinct Tiger shark fossil species †*Galeocerdo mayumbensis* to show several diagnostic features, including a wide triangular crown with an oblique apex; the lesser concave distal cutting edge rather than the other members of the genus *Galeocerdo*; the presence of coarse serrations with secondary serration on the distal cutting edge; a deep root with strong arcuate ventral edge and without transverse groove [21,52,53].

†*Galeocerdo mayumbensis* was originally described from the Miocene beds of Western Africa, however, the current distribution also includes Miocene outcrops from Brazil, Colombia, Madagascar, and Libya [23,31,53,54]. It is assumed that some *Galeocerdo* specimens found in Miocene outcrops from the USA, Panama, Brazil, and India could be synonyms of †*G. mayumbensis* [31]; however, the revision of these materials is still necessary to corroborate this argument. The specimen described herein is the first undoubtedly evidence of †*G. mayumbensis* in outcrops from the Western North Atlantic, confirming the hypothesis that this species was widely distributed in marine shallow environments during the Miocene [31].

Family Hemigaleidae Hasse, 1879 [55]

Genus *Hemipristis* Agassiz, 1835 [56]

†*Hemipristis serra* Agassiz, 1835 [56]

(Fig 6 E-H)

Referred specimen

IGM 12312; an incomplete lower jaw tooth with the root almost completely damaged. The specimen was collected in the cenote X-Nabuy.

Description

The incompleteness of the unique tooth found does not preclude determining its total length and depth. The best-preserved portion is the crown, which is deeper than large and presents a small lateral constriction at the midpoint (Fig E-F). Both cutting edges have small serrations on the apical portion, which is discontinued in the base of the crown. The shoulders are very small and present a few minutes of serrations. Only a little central foramen is visible on the root.

Remarks

Hemipristid (Snaggletooth sharks) upper jaw teeth are remarkable due to the curvature and coarse serrations in the mesial and distal upper jaw cutting edges [21]. Nevertheless, the lower teeth in †*Hemipristis serra* are different: the crown is narrow, laterally sigmoid, and showing the cutting edges finely serrated and straight; the cutting-edge serrations are discontinued close to the base of the crown; the shoulder is small and is ornate with a few small serrations [21,39]. Although not complete, the unique specimen here is considered as †*H. serra* due to the narrow and straight crown and the presence of discontinued serrations on the cutting edges. This species was cosmopolitan during the Miocene-Pliocene [54], and its distribution in Mexico ranged from the Middle Oligocene to the Pliocene strata [34,35]. The unique extant species of the genus is *Hemipristis elongata* [57], which inhabits inshore and offshore on the continental and insular shelves in the Indo-West Pacific from 1 to 30 meters [21,30].

Order Lamniformes Berg, 1937 [58]

Family †Otodontidae Glikman, 1964 [59]

Genus †*Otodus* Agassiz, 1838 [60]

†*Otodus* (*Carcharocles*) *megalodon* Agassiz, 1843 [60]

(Fig 7 A-P)

Fig 7. †*Otodus* (*Carcharocles*) *megalodon* Agassiz, 1843 [51] specimens (A to P; IGM 12316, IGM 12315, IGM 12313, and IGM 12317) and *Carcharodon carcharias* Linnaeus, 1758 [61] (Q to T; IGM 12314) from Carrillo Puerto Formation.

(A, E, I, M, and Q) Labial view. (B, F, J, N, and R) Lingual view. (C, G, K, O, and S) Right lateral view. (D, H, L, P, and T) Left lateral view. The scales indicate 10 mm.

Referred material

IGM 12313; an incomplete tooth where the root is lacking, and the crown shows a lateral cusplet only on the left side. IGM 12315; an incomplete tooth, where the root and the distal cutting edge are not complete. IGM 12316; a complete tooth with the apex of the crown slightly turned to the left side. IGM 12317; an almost complete tooth with a small crown depth and a large root length, the crown is slightly turned to the left, and the distal root lobe is lacking. The specimens IGM 12313 to IGM 12316 are from the cenote San Juan and IGM 12317 is from the cenote Sambulá.

Description

There are four teeth in distinct degrees of preservation. The total length ranges from 42 to 49 mm, and the total depth from 47 to 56 mm in complete specimens. In general, the teeth are labio-lingually flattened, and the crown is triangular, with the apex not labially projected. There is a notable high degree of heterogeneity in the crow shape: the labial surface is straight, and the lingual surface is slightly concave except in IGM 12315 and IGM 12316, which the lingual surface is straight and inclined; in IGM 12316 and IGM 12317, both mesial

and distal cutting edges are concave; however, the mesial cutting edge in IGM 12313 is convex in the apical portion and concave at the base, while the distal edge is concave along all the border. In the specimen IGM 12315, only the mesial cutting edge is present and is convex. In IGM 12317, the crown is shallower and thinner than the others. Cusplets are present over the distal root lobe in IGM 12313 and the mesial lobe in IGM 12317. The cusplet shape is different in each specimen: in IGM 12313, the cusplet is separate from the crown by a deep notch and presents coarse serrations with a robust medial projection, while in IGM 12317, the cusplet is almost continuous with the cutting edge, the serrations are lesser developed, and the medial projection is lacking. The medial portion of the ventral edge of the crown in lingual view is deep and angular, forming a large triangular neck, which is best observed in IGM 12316 and IGM 12317. The root is deep and ventrally arcuate. The root lobes are robust with circular edges. The lingual protuberance of the root is pronounced. The central foramen is reduced to small circular perforations and the transverse groove is lacking.

Remarks

The specimens are considered †*Otodus* (*Carcharocles*) *megalodon* due to the following combination of features: teeth labio-lingually flattened and single cusped; cutting edges serrated and not notched; dental bands triangular and well developed; root notched and protruding lingually at midline of the tooth; transverse groove absent in lingual root protrusion; basal margin of the enamel of lingual face gently arched. The small length indicates that all teeth belonged to a juvenile shark. The presence of cusplets should indicate co-occurrence of the species †*Otodus* (*Carcharocles*) *chubutensis* or †*Otodus*

(*Carcharocles*) *angustidens*; however, the cusplet present in one cutting edge is a general feature found in juveniles [48,62] that should persist in some adult forms [63].

†*Otodus* (*Carcharocles*) *megalodon* is a worldwide distributed shark during the Miocene and Pliocene, with the lack of occurrence only on the polar seas [48]. In Mexico, Megalodon sharks are reported on Miocene and Pliocene outcrops from Baja California (Trinidad and Tirabuzón formations) and on Miocene outcrops from Palenque, Chiapas (Tulijá Formation) [34,35,64].

Family Lamnidae Berg,1958 [65]

Genus *Carcharodon* Smith in Müller & Henle, 1838 [66]

Carcharodon carcharias Linnaeus, 1758 [61]

Referred material

IGM 12314, an incomplete and small tooth, where the root is lacking. The specimen is from the cenote San Juan.

Description

The teeth are flattened labio-lingually, and only the crown and a small portion of the root are preserved. The crown is narrow and triangular, with the apex straight. The labial surface of the crown is almost straight, while the lingual surface is medially concave. There

is a shallow curvature of the tip of the crown to the labial portion. Both mesial and distal cutting edges have a shallow notch close to the base of the crown and are ornate with strongly coarse serrations. The serrations are uniform along the entire surface. Cusplets are lacking. The medial curvature at the base of the labial surface is shallow. The distance between the base of the mesial cutting edge and the ventral edge of the crown forms an angle of about 13 degrees. The root is poorly preserved; only a small portion of the mesial root lobe is observed.

Remarks

This unique tooth studied here is assigned to the White shark due to the absence of a triangular and acute neck on the lingual surface, the coarser serrations along the cutting edges, and more labio-lingually compressed teeth in comparison with †*Otodus* (*Carcharocles*) *megalodon*. Although some hypothesis indicates that these species should have an allopatric distribution [39], there is the first report of the co-occurrence of both species in the late Pliocene strata of Baja California, Mexico [62]. Our evidence indicates that both species occurred on both sides of the Mexican coasts during the Miocene-Pliocene.

Division Bathomorphi Cappetta, 1980 [67]

Order Myliobatiformes Compagno, 1973 [26]

Family Myliobatidae Bonaparte, 1838 [24]

Genus *Pteromylaeus* Garman, 1908 [68]

Pteromylaeus sp.

(Fig 8 A-L)

Fig 8. *Pteromylaeus* (IGM 12319 and 12320) and *Rhinoptera* (IGM 12321) specimens from Carrillo Puerto Formation.

(A, G, and M) Occlusal view. (B, H, and N) Ventral view. (D, J, and P) Lingual view. (E, K, and Q) Labial view. (C, I, and O) Right lateral view. (F, R, and L) Left lateral view. The scales indicate 5 mm.

Referred material

IGM 12319; an almost complete tooth, with the crown eroded. The tooth is completely black. IGM 12320; a broken tooth exhibiting only about one-third of the total length. The teeth are black with the base of the root yellowish. Both specimens are from the cenote X-Nubay.

Description

The teeth are hexagonal and horizontally enlarged. The best-preserved specimen is 21.7 mm in length and 4.9 mm in depth in occlusal view. Only a little remnant of the crown is preserved in all specimens. The surface of the crown is rough and slightly tacky. The labial overhang is strongly pronounced, with the external labial edge slightly concave in IGM

12319. The lingual ledge is not pronounced and is strongly enameled, with a distinct brown coloration in both specimens studied. Close to the upper surface of the polyaucorhyzus root is a horizontal series of perforations (Fig 8 E and K). The loblets are observed as several deep grooves on the ventral view. The loblets of the lateral extremities are triangular.

Remarks

These two teeth are considered to belong to the genus *Pteromylaeus* due to the presence of foramina well-developed on both labial and lingual sides and a deep polyaucorhyzus root with a weak lingual inclination [53]. The preservation of the few teeth described here does not allow us to determine the specific taxonomic rank of these fossils. *Pteromylaeus'* fossil record includes strata from the Miocene of France [69], Miocene-Pliocene from Libya [53], and the Pungo River, in North Carolina, USA [21]. The small length of these tooth plate fragments indicates that these specimens are juveniles. Currently, the only one species known, *P. bovinus* [70], is distributed in the eastern Atlantic and the Mediterranean Sea, ranging from 10 to 100 meters from the coast [21,71].

Family Rhinopteridae Jordan & Evermann, 1896 [72]

Genus *Rhinoptera* Cuvier, 1829 [73]

Rhinoptera sp.

(Fig 8 M-R)

Referred specimen

IGM12321; an incomplete small tooth plate with light brown coloration and the crown eroded. The specimen is from the cenote Sambulá.

Description

Unique small teeth are hexagonal and horizontally enlarged. The teeth are 13.8 mm in length and 3.9 mm in depth in the occlusal view. Like other stingray specimens found, only a little remnant of the crown is preserved. The labial overhang is less pronounced than the *Pteromylaeus* tooth, and the external labial edge is straight. The lingual ledge is not pronounced and is strongly enameled, with the same brown coloration as the other regions of the teeth. The foramina at the dorsal surface close to the polyaucorhyzus root is vestigial and better visualized at the lateral extremities (Fig 8 O and R). The root loblets are not deep like in the *Pteromylaeus* specimens observed and seem to be further away from each other (Fig 8 N). The loblets of the lateral extremities of the root are triangular.

Remarks

The unique specimen herein described is considered a member of the genus *Rhinoptera* due to the absence of foramina on both labial and lingual sides of the tooth and the shallow and not uniform polyaucorhyzus with a lower number of grooves on the root in comparison with specimens belonged to the genus *Pteromylaeus*. The genus *Rhinoptera* was widely distributed along marine environments around the globe during the Miocene-Pliocene. In Mexico, *Rhinoptera* species are reported from the Oligocene outcrops of the San

Gregorio y El Cien Formations, and Miocene strata of the San Izidro Formation, both in Baja California [34,35]. The tooth is small, indicating that the specimen studied is a juvenile.

Infraclass Teleostei Müller, 1845 [74]

Series Eupercaria Betancur-R. *et al.*, 2013 [75]

Order Tetraodontiformes Berg, 1937 [58]

Family Diodontidae Bonaparte, 1835 [76]

Genus *Chilomycterus* Brisout de Barneville, 1846 [77]

†*Chilomycterus dzonotensis* sp. nov.

(Figs 9 and 10)

Fig 9. Photography of the premaxilla of †*Chilomycterus dzonotensis* sp. nov. (IGM 12322).

(A) Ventral view. (B) Frontal view. (C) Dorsal view. (D) Posterior view. (E) Right lateral view. (F) Left lateral view. The scale bars indicate 10 mm.

Holotype

IGM 12322; a premaxilla with 16.5 mm in length in frontal view. The premaxilla of both sides fuses in a unique element, in which the portion comprising the biting edge is broken (Fig 9 A and C). The posterior bone elements, including the internal tooth plate, and the posteromedial process of the premaxilla are well-preserved. The specimen is from the cenote X-Nabuy.

LSID.

Diagnosis

†*Chilomycterus dzonotensis* sp. nov. differs from the other living and fossil specimens of the genus due to the presence of a concavity in the external lateral edges of the tooth plates that composes the occlusal crushing surface, which in this species is formed by eight quadrangular and paired teeth, with the anterior pair being the largest teeth of the series. The tooth plate gradually narrows laterally until the penultimate pair of teeth. The last pair is large, like the first. Other features that distinguish †*C. dzonotensis* from the other taxa are the absence of teeth embedded into the bone matrix of the premaxilla, and the lack of fusion of the upper jaw bones.

Derivation of the name

The specific name is the Latinization of the Mayan word "*d'zonot*", which means cave with water and the word which the Mayan people referred to the sinkholes. The generic and specific name means "the *Chilomycterus* species which belongs to a cenote".

Description

The upper jaw is massive, with the premaxilla fully fused to its opposite in the midline. The anterior surface of the premaxilla has several irregular lines formed by small bony protuberances. In the fusion point of the premaxilla, there is a posteromedial process which in extant diodontid species articulates by fibrous tissues with the palatine and the ethmo-vomer (Tyler, 1980; 346) [22]. The oval lateral process on each side of the premaxilla should be the premaxillary articular process, a widespread feature found in acanthomorphs. The alveolar process of the premaxilla composes the posterior portion of the biting edge.

There is a lateral perforation on each side of the premaxilla and at least seven perforations in the deep gap between the biting edge and the anterior surface of the premaxilla. The external perforations are well-developed and irregular in shape (i.e., some are circular, while others are oval). The posterior perforations are small and circular (Fig 9).

The biting edge comprises several vertical series of teeth. Different from some other *Chilomycterus* species (Tyler, 1980; 365) [22], the teeth of the biting edge are not embedded in the bone matrix. The teeth are flat, circular in shape, and organized in vertical series. The teeth' vertical series are located laterally in the biting edge and are reduced in number, comprising about two or three teeth. The teeth of the vertical series gradually increase to the medial portion of the premaxilla, where the vertical series has seven teeth. This conformation forms a triangular and robust beak used to scrap the food.

The interior of the premaxilla comprises a series of eight plates accommodated in pairs. These plate pairs overlap each other, and only the posterior edges are externally visible,

forming the internal trituration plate. Like the biting edge, the trituration plates in †*Chilomycterus dzonotensis* sp. nov. are different from other diodontid species since all plates are exposed while (Fig 9 A and E), in general, they are embedded in the premaxilla (see Tyler, 1980; 365, for example) [22].

In the ventral view, it is possible to observe the external portion of the pair of plates that compose the trituration portion of the upper jaw. There is a space of about 1.7 mm between the biting edge and the trituration plate. From this viewpoint, the plates are almost rectangular and cover most of the ventral surface of the upper jaw, reaching the posterior border of the premaxilla. On the ventral surface, it is also possible to note that the plates are strongly articulated laterally and are not equidistant or parallel. At the portion of the internal lateral articulation, the first four anterior plates are ventrally projected. The posterior border is rounded. The length of the last pair of teeth in the ventral view is equal to the first two, forming a concave external lateral surface of the trituration plate.

The medial surface of the teeth series that composes the trituration plate in diodontids is separated into smooth and papillae portions. Like in †*Chilomycterus ferrerai* (Aguilera et al., 2017, Figure 4) [23], †*C. dzontensis* shows the papillae portion very pronounced. The papillae portion in the species herein described reaches almost the anterior border of the first. The smooth surface of the posterior trituration plates is larger than in other *Chilomycterus* species.

Remarks

The family Diodontidae (the porcupine fishes or burrfishes) comprises a total of seven genera, with 18 living and 14 valid fossil (and several unidentified) species distributed in marine environments of the Atlantic, Indian, and Pacific oceans [22,23,78,79]. The species †*Chilomycterus dzonotensis* sp. nov. is considered a diodontid due to the presence of the premaxilla fully fused to their opposite member, the marginal massive incisor-like teeth (the biting edge) and the occlusal crushing surface (the trituration plate) in the premaxilla, and the maxilla with a deeply indented surface (Fig 9 A-C).

The jaw elements are the most common bone structures preserved in diodontid fossils. The family has at least 62 ancient records [80] with the oldest report belonging to the Late Cretaceous strata from Brazil, precisely in the Maastrichtian strata from Gramame Formation, Paraiba state [81]. The species herein described is easily differentiated from the Mesozoic Brazilian diodontid due to the reduced number of internal plates in the premaxilla (see Gallo et al. [81], Figure. 2).

The diodontid diversification is notable during the Paleogene, with several genera described such as †*Heptadiodon* Bronn, 1855 [82], †*Eodiodon* Casier, 1952 [83], †*Progymnodon* Dames, 1883 [84], †*Prodiodon* Le Danois, 1955 [85], †*Zignodiodon* Tyler and Santini, 2002 [86], †*Pshekhadiodon* Bannikov and Tyler, 1997 [87], and †*Oligodiodon* Sauvage 1873 [88]. Except for †*Heptadiodon* and †*Zignodon fornasieroae* Tyler and Santini, 2002 [86], which the information provided about the upper jaw is scarce to compare with †*C. dzontensis,* the other Paleogene taxa can be distinguished from the species herein described.

The genus †*Eodiodon* is the unique diodontid genus where the tooth plate is lacking ([83], Figure 1A); the genus †*Progymnodon* has tooth plates almost in contact with the biting

edge and the tooth plates are semicircular ([83], Figure 1B); ([89], Plate I, Figure 1). The species †*Psekhadiodon parini* Bannikov and Tyler 1997 [87] have the first pair of trituration plates large such as is found in the species described; however, there are only four pairs of tooth plates in the premaxilla, and the external lateral edges of the plates are convex. Inside the genus †*Prodiodon*, only †*P. tenuispinus* has the internal tooth plate of the lower jaw preserved. These plates are parallel and gradually increase in length ([86], Figure 28).

During the Neogene, the generic diodontid diversity reduced; however, it was the moment when species of extant genera rose. Inside the Cenozoic groups, the genus †*Oligodiodon* is unique to pass through the Paleogene-Neogene boundary [90]. Like †*C. dzonotensis* sp. nov., †*Oligodiodon* have at least seven pairs or trituration plates (Fig 10); however, the teeth are strongly imbricated, and the width of the plates is equal along all the occlusal surfaces (Fig 10A). In †*Chilomycterus dzonotensis* sp. nov., the trituration plates are not strongly imbricated, and the plate width is unequal, with the external lateral surface forming a constriction in the occlusal surface (Fig 10E).

Fig 10. Schematic drawing of the premaxilla from different Miocene diodontid species.

(A) †*Oligodiodon* sp. (modified from Schultz [90]). (B) †*Chilomycterus ferrerai* (Santos and Travassos, 1960) [91]. (C) †*C. vetus* (Leidy, 1877) [92]. (D) †*Diodon serratus* Aguilera, Carrillo-Briceño and Rodriguez, 2017 [23]. (B-D) modified from Aguilera et al. [23]). (E) †*C. dzonotensis* sp. nov (IGM 12322). The scale bars indicate 5 mm.

The differentiation of fossil species of extant genera is based mainly on the position of the trituration internal plates about the biting edge. According to Aguilera et al. [23], the crushing plates of *Diodon* are on the posterior portion of the occlusal surface, whereas in *Chilomycterus,* the internal plates are close to the biting edge. Adult species are also different in the number of internal plates. Large *Diodon* species has about 35 trituration plates, while the adult *Chilomycterus* has only 18 plates [22,23].

The length of these structures in the species herein studied indicates a juvenile specimen with about 10 cm ([22], Figure 302; [23], S1 Figure); therefore, taxonomic assignment is not possible through the number of trituration plates. Nevertheless, the approximation of the trituration tooth plate with the biting edge (see Fig 9 and [23], Figure 4), the alternate position of the tooth plates, and the teeth conformation in cross-section (see Fig 10 and [22], Figures 294 to 300) suggest that the species herein described belongs to the genus *Chilomycterus*.

The genus *Chilomycterus* is the most speciose in the diodontid fossil record, especially in the Proto-Caribbean region during the Miocene [23]. The American *Chilomycterus* fossil record includes †*C. circunflexus* Leriche, 1942 [93] from La Habana, Cuba, and form Florida, USA; †*C. exspectatus* Aguilera, Carrillo-Briceño, and Rodriguez, 2017 [23], †*C. tyleri* Aguilera, Carrillo-Briceño, and Rodriguez, 2017 [23,94], †*C. gatunensis* Toula, 1909 [94], from Gatun Formation, San Judas Tadeo, Colon, Panama; †*C. kugleri* Casier, 1958 [95] from Gross Morne Formation, and †*C. vetus* Leidy, 1877 [92] from Tamana Formation, both from Trinidad; †*C. ferrerai* Santos & Travassos, 1960 [91], from several localities of the Pirabas Formation, at Para State, Northern Brazil, and from the Canture Formation, Venezuela; one unidentified *Chilomycterus* species from Tuira

Formation, Panama; and other unidentified species from Guajira Peninsula, Colombia [23]. No living or fossil *Chilomycterus* species possess constriction on the external edge of the occlusal crushing surface ([23]; Figures 4-8), which allows us to consider that this fossil represents a new *Chilomycterus* species.

Although diodontids were very diverse during the Neogene in the tropical region comprising the northern portion of South American, Central, and North America ([23], Figure 9), there is the first diodontid fossil record from Miocene-Pliocene strata from the Gulf of Mexico.

Discussion

The fossil diversity of the Carrillo Puerto Formation

The fossil diversity from the Carrillo Puerto Formation is poorly known. The only three vertebrate species formerly cataloged and housed in a paleontological collection are a small cervid (IGM 4568) and two aquatic mammal species, †*Xenosiren yucateca* Domning, 1989 [16] and †*Corystosiren varguezi* Domning, 1990 [17], both belonging to the family Dugongidae and the subfamily Rytiodotinae [16,17] (Table 1). The description of †*Xenosiren yucateca* is from two bone fragments (IGM 4190 and IGM 4191) found in the north portion of the cenote Kambul, about 15 km northwest of Merida, state of Yucatan (IGM-loc. 2397); while †*Corystosiren varguezi* is described from a nearly complete skull, vertebrae remain, and ribs (IGM 4569) found exposed in ground surface on Rancho Chapas, close to Tizimin municipality, northeast Yucatan (IGM-loc. 2398).

Table 1. Vertebrate fossil record reported or mentioned from the Carrillo Puerto Formation

Taxon	Catalog number	Reference
Carcharhinus brachyurus	IGM 12301, IGM 12302, IGM 12303, IGM 12318	This study
Carcharhinus leucas	IGM 12304, IGM 12305, IGM 12306, IGM 12307	This study, Domning (1990)
Carcharhinus macloti	IGM 12308	This study
Carcharhinus perezii	IGM 12309	This study
Carcharhinus sp.	IGM 12310	This study
Galeocerdo sp.	-	Domning (1989)
Galeocerdo aff. *rosaliensis*	-	Domning (1990)
Galeocerdo mayumbensis	IGM 12311	This study
Hemipristis serra	IGM 12312	Domning (1989), this study
Isurus hastalis	-	Domning (1989, 1990)
Otodus (*Carcharocles*) *megalodon*	IGM 12313, IGM 12315, IGM 12316, IGM 12317	Domning (1989, 1990), this study
Carcharodon carcharias	IGM 12314	This study
Pteromylaeus sp.	IGM 12319, IGM 12320	This study
Rhinoptera sp.	IGM 12321	This study
Chilomycterus dzonotensis sp. nov.	IGM 12322	This study
Corystosiren varguezi	IGM 4569	Domning (1990)
Xenosiren yucateca	IGM 4190	Domning (1989)
Crocodilia undetermined	-	Domning (1990)

Cervidae undetermined.	IGM 4568	Domning (1989)

On limestones from Cenote Noc Ac and the type-locality of †*Corystosiren varguezi* mentioned the presence of other fossil organisms, including invertebrates, shark teeth, and crocodilian fragments [16,17]. The shark species include *Carcharhinus leucas*, †*Otodus* (*Carcharocles*) *megalodon*, *Isurus hastalis*, *Hemipristis serra*, a tiger shark related to †*Galeocerdo rosaliensis* Applegate, 1978, and one more advanced than †*G. aduncus* [17]. According to the author, these taxa were described by Shelton P. Applegate [17]; however, the catalog number, the current location of the specimens, and the morphological evidence that support the taxonomic attributions are unknown (Table 1). Our study corroborates the presence of *C. leucas* and †*O.* (*C.*) *megalodon*, and *H. serra*. The presence of *I. hastalis* is not confirmed and a distinct *Galeocerdo* species (†*G. mayumbensis*) is determined. Furthermore, five other shark taxa and two stingrays are reported to compose the chondrichthyan diversity of the Carrillo Puerto Formation (Table 1).

We also recognize a new teleost fish from a small upper jaw, which presents the premaxilla anteriorly fused and two separated tooth plates; enough features to classify it as a member of the family Diodontidae. The crushing occlusal tooth plate shows similarities with the genus *Chilomycterus*; however, the presence of a posterior constriction and all teeth exposed are conditions not observed in other fossil or extant diodontid species. This finding represents an increment in the diversity and distribution of the family Diodontidae during the Miocene-Pliocene in the Western Atlantic Sea domains.

Fish diversity significance

Previous studies based on the lithological features and fossil invertebrates indicated a shallow shelf depositional environment with internal neritic conditions and middle neritic influence on the Carrillo Puerto Formation [14]. Comparing the distribution of the Carrillo Puerto Formation with the Eocene Chichén Itzá Formation and the current littoral composition of the Yucatan Peninsula in present-day is evident a clear marine regression during the Cenozoic on this region (Fig 1). The marine regression provided large periods of warm shallow marine water associated with a coral reef system; an excellent refuge to small organisms, such as juvenile teleost specimens, young sharks, and stingrays.

Our results are in complete accordance with the paleoenvironmental interpretation for the Carrillo Puerto Formation since all taxa are marine species that usually inhabit warm shallow waters not exceeding 350 meters in depth, and include reef-associated species, such as *Carcharhinus brachyurus* and *C. perezii*. Remarkably, both chondrichthyans and teleost fossil fish remains found in the sinkholes of the Carrillo Puerto Formation are very small. The upper jaw of †*Chilomycterus dzonotensis* sp. nov., for example, has only 16.5 mm in horizontal length, and the shape and number of the tooth plates in the occlusal surface indicate that the total length of the specimen should be about 10 cm [22]. The biggest Megalodon tooth is about 56 mm in depth. The predominance of small organisms is a strong indicator that the deposition environment of the Carrillo Puerto Formation was a shelter for sub-adult individuals.

Another feature that reinforces the predominant presence of juveniles on the Carrillo Puerto Formation strata is the observation of lateral cusplets in two †*Otodus* (*Carcharocles*) *megalodon* teeth. The presence of lateral cusplets was initially proposed to separate the species †*Otodus* (*Carcharocles*) *chubutensis* and †*O.* (*C.*) *angustidens* from *O.* (*C.*)

megalodon. Nevertheless, recent studies indicate that this is a common feature found in juvenile specimens of Megalodon Sharks [48]. The predominance of juvenile species indicates the presence of a paleo-nursery in the Gulf of Mexico, such was previously reported in other Miocene-Pliocene fossil localities in Central America [48].

Another interesting point to consider with the revelation of the fish fauna from Carrillo Puerto Formation is the repeated co-occurrence of the White Shark and Megalodon on Mexican coasts during the Miocene-Pliocene. Traditionally is believed that *Charcharodon carcharias* and †*Otodus* (*Carcharocles*) *megalodon* have had allopatric distributions [39]; however, the co-occurrence of these species was previously reported from Miocene outcrops of North Carolina and Peruvian localities [21], for example. In Mexico, this co-occurrence is reported from the Pliocene sediments of Baja California [21,62]. Our results reinforce the co-occurrence of White and Megalodon Sharks in America during the Miocene-Pliocene, with a posterior extinction of the last species.

Like Megalodon sharks, the extinction of other taxa in American marine environments after the Miocene-Pliocene is observed in the genus *Pteromylaeus* and the shark species *Carcharhinus macloti*. Different from Megalodon which was completely extinguished after the Pliocene, extant organisms of *Pteromylaeus* and *C. macloti* are currently distributed in marine shallow habitats of tropical and temperate regions, but not on the North Atlantic coasts of America [46]. The fossil record on the Carrillo Puerto Formation reinforces the presence of these species on the Western Atlantic coast during the Miocene-Pliocene and is a strong indicator of the loss of habitat of some species from local extinctions. A more detailed study is necessary to understand the nature of these extinctions after the Miocene-Pliocene; however, is probable that the repetitive glaciation events that occurred at

that time [96] should be related to the extinction and reduction of distribution of some taxa on Atlantic coasts during the Late Cenozoic.

Conclusion

The underwater strata from the Carrillo Puerto Formation inside the sinkholes are remarkable evidence of the gradual marine regression that occurred during the Late Cenozoic that was responsible for the formation of the current Yucatan Peninsula. Here, we show the fish fauna that inhabited this environment and reveal biodiversity composed of extinct taxa, such as the Megalodon shark and the new burrfish species †*Chilomycterus dzonotensis* sp. nov.; extant organisms that are currently distributed in marine Mexican coasts, such as the White shark; and extant species that are not currently distributed on western Atlantic domains, such as the Hardnose shark and the Bull ray.

The fish fauna discovered represents an important increment in vertebrate diversity during the Late Miocene and Early Pliocene of the Gulf of Mexico since several described taxa had not been previously reported. Although some shark species were previously mentioned in the Carrillo Puerto Formation [16,17], the specimens that were used for the identification are lost and not cataloged. Therefore, the organisms shown here are the unique physical examples formally deposited in a public paleontological collection for future investigations.

The fossil diversity corroborates the shallow marine paleoenvironment associated with a coral reef system for the Carrillo Puerto Formation. Furthermore, the small size of all

specimens studied, together with the presence of cusplets in Megalodon shark tooth indicates the presence of a shelter, or paleo-nursery, for elasmobranchs and teleost on the depositional sediments that compose the Carrillo Puerto Formation today. Finally, the presence of species that currently are not distributed in the Gulf of Mexico points out local extinctions during the Late Cenozoic on the Northwestern Atlantic, probably associated with glaciation events.

Acknowledgments

We are grateful to the authorities responsible for the Cenotes and the *Secretaría de Desarrollo Sustentable* (SDS) of Yucatan, and the *Subdirección de Arqueología Subacuática* (SAS) of the *Instituto Nacional de Antropología e Historia* (INAH) for providing permits to collect material. Thanks to Pablo Bayardo Gómez (Overhead Diving Mexico) and Michael Silva Netto (CCRXTREME) for cave dive support. Thanks to Myriam Miranda and Lourdes Fernández for the material transport. Thanks to Jesús Alvarado Ortega and Violeta Amparo Romero Mayén for providing information about the previously described species from the Carrillo Puerto Formation, and for cataloging and accommodating the specimens studied in the National Collection of Paleontology of Mexico, UNAM. Thanks to the reviewers for the improvement in the final version of the manuscript. The present work is part of the Underwater Archaeological Atlas project, coordinated by Helena Barba Meinecke from SAS-INAH.

References

1. USEPA. A Lexicon of Caves and Karst Terminology with Special Reference to Environmental Karst Hydrology. U. S. Environmental Protection Agency, editor. Office of Research and Development, National Center for Environmental Assessment, Washington, DC, EPA/600/R-02/003, 2002.; 2002.

2. Kambesis PN, Coke I V. The Sac Actun System. Boletín Geológico y Minero. 2016;127: 177–192.

3. Angyal D, Chávez-Solís EM, Liévano-Beltrán LA, Magaña B, Simoes N, Mascaró M. New distributtion records of subterranean crustaceans from cenotes in Yucatan (Mexico). Zookeys. 2020;911: 21–49.

4. Schmitter-Sotto JJ, Escobar-Briones E, Alcocer J, Suárez-Morales E, Elías-Gutiérrez M, Marín L. Los cenotes de la Península de Yucatán. In: De la Lanza-Espino G, García-Calderón JL, editors. Lagos y presas de México. México D.F.: AGT; 2002. pp. 337–381.

5. Bauer-Gottwein P, Gondwe BRN, Charvet G, Marín L, Rebolledo-Vieyra M, Merediz-Alonso G. Review: The Yucatán Peninsula karst aquifer, Mexico. Hydrogeol J. 2011;19: 507–524. doi:10.1007/s10040-010-0699-5

6. Arcega-Cabrera F, Sickman JO, Fargher L, Herrera-Silveira J, Lucero D, Oceguera-Vargas I, et al. Groundwater Quality in the Yucatan Peninsula: Insights from Stable Isotope and Metal Analysis. Groundwater. 2021;59: 878–891.

7. Perry E, Velázquez-Oliman G, Marín L. The hydrogeochemistry of the karst aquifer system of the Northern Yucatan Peninsula, Mexico. Int Geol Rev. 2002;44: 191–221.

8. QRSS. Underwater cave survey in Quintana Roo. Quintana Roo Speleological Survey, editor. Quintana Roo Speleological Survey. 2023. 30 Oct 2023. Available from: https://legacy.caves.org/project/qrss/qrss.htm

9. Rissolo D. Ancient maya cave use in the Yalahu region, Northern Quintana Roo, Mexico. Association for Mexican Cave Studies. Bulletin 12, editor. Austin Texas: Association for Mexican Cave Studies; 2003.

10. Arroyo-Cabrales J, Luna P, Chatters J, Rissolo D, Chávez-Arce R, Nava-Blank A, et al. Underwater Archaeology and Prehistory: The Case of the Cenotes in Mexico. In. World Heritage Human Origin Sites and the World Heritage Convention in the Americas. Heads 5, Volume 1. World Heritage Human Origin Sites and the World Heritage Convention in the Americas Heads 5, Volume 1. United Nations Educational, Scientific, and Cultural Organization, World Heritage Convention; 2015. pp. 54–60.

11. López-Martínez RA, Barragán R, Rosado-González E. Sac Actun Underwater Cave System. In: International Union of Geological Sciences, editor. The first 100 IUGS International Commission on Geoheritage. International Union of Geological Sciences; 2022. pp. 260–261.

12. Bonet F, Butterlin J. Reconocimiento Geológico de la Península de Yucatán: Enciclopedia Yucatense. Gobierno del Estado de Yucatán, editor. Mérida, Yucatán: Gobierno del Estado de Yucatán; 1959.

13. Butterlin J, Bonet F. Las Formaciones Cenozoicas de la parte mexicana de la Península de Yucatán: México. Instituto de Geología UNAM. 1963; 1–43.

14. SGM. Formación Carrillo Puerto. Léxico Estratigráfico de México, Servicio Geológico Mexicano, editors. 2011.

15. SGM. Carta Geológico-Minera Estados de Campeche, Quintana Roo y Yucatán, escala 1:50000. Servicio Geológico Mexicano, editor. 2006. 30 Oct 2023. Available from: https://www.sgm.gob.mx/GeoInfoMexGobMx/#

16. Domning DP. Fossil Sirenia of the West Atlantic and Caribbean region. III. Xenosiren yucateca, gen. et sp. nov. J Vertebr Paleontol. 1989;9: 429–437.

17. Domning DP. Fossil Sirenia of the West Atlantic and Caribbean region. IV. Corystosiren varguezi, gen. et sp. nov. J Vertebr Paleontol. 1990;10: 361–371.

18. Miranda-Huerta A. Carta Geológico-Minera Tizimín F16-7 Escala 1:250,000 Estado de Yucatán. Servicio Geológico Mexicano. 2005; 1–39.

19. Zarate-Barradas RG, Lemus-Bustos O, Miranda-Huerta A, Cureño-Suriano R. Carta Geológica-Minera y Geoquímica Mérida, Clave F16-10, Escala 1:250,000, Estados de Yucatán, Campeche y Quintana Roo. Servicio Geológico Mexicano. 2005; 56p.

20. López-Ramos E. Estudio Geológico de la Península de Yucatán. Boletín Asociación Mexicana de Geólogos Petroleros. 1983;25: 23–76.

21. Purdy RW, Schneider VP, Applegate SP, McLellan JH, Meyer RL, Slaughter R. The Neogene sharks, rays, and bony fishes from Lee Creek Mine, Aurora, North Carolina. In: Ray CE, Bohaska DJ, editors. In Geology and Paleontology of the Lee Creek Mine, North Carolina, III. Smithsonian Contributions to Paleobiology; 2001. pp. 71–202, 84 fig., 1 tabl.

22. Tyler JC. Osteology, phylogeny and higher classification of the fishes of the Order Plectognathi (Tetraodontiformes). NOAA Tech Rep NMFS. 1980; Circular 434.

23. Aguilera O, Silva GOA, Lopes RT, Machado AS, dos Santos TM, Marques G, et al. Neogene Proto-Caribbean porcupinefishes (Diodontidae). PLoS One. 2017;12: e0181670. doi:10.1371/journal.pone.0181670

24. Bonaparte CL. Selachorum tabula analytica, Systema Ichthyologicum. Mémoires de la Société Neuchâteloise des Sciences Naturelles. 1838;2: 1–16.

25. Cope ED. Observations on the systematic relations of the fishes. American Naturalist. 1871;5: 579–59.

26. Compagno LJV. Interrelationships of living elasmobranchs. Zool J Linn Soc. 1973;53: 15–61.

27. Jordan DS, Evermann BW. Description of six new species of fishes from the Gulf of Mexico, with notes on other species. Proceedings of the United States National Museum. 1886;9: 466–476.

28. Blainville H. Prodrome d'une nouvelle distribution systématique du règne animal. Bulletin des Sciences, par la Société Philomathique de Paris. 1816;8: 105-112 [sic for 113-120] + 121-124.

29. Günther A. Catalogue of the fishes in the British Museum. London, British Museum (Natural History). 1870;8: 549 p.

30. Compagno LJV. FAO Species Catalogue. Vol 4: Sharks of the world, Part 2 - Carcharhiniformes. FAO Fisheries Synopsis No 125. 1984;4: 251–633.

31. Carrillo-Briceño JD, Luz Z, Hendy A, Kocsis L, Aguilera O, Vennemann T. Neogene Caribbean elasmobranchs: diversity, paleoecology and paleoenvironmental significance of the Cocinetas Basin assemblage (Guajira Peninsula, Colombia). Biogeosciences. 2019;16: 33–56.

32. Carrillo-Briceño JD, Aguilera OA, de Gracia C, Aguirre-Fernández G, Kindlimann R, Sánchez-Villagra MR. An Early Neogene Elasmobranch fauna from the southern Caribbean (Western Venezuela). Palaeontologia Electronica. 2016;19.2.27A: 1–32.

33. Medeiros S, Oddone MC, Francischini H, Diniz D, Dentzien-Dias P. Quaternary fossil shark (Neoselachii: Galeomorphii and Squalomorphii) diversity from southern Brazil. J South Am Earth Sci. 2023;122.

34. González-Rodríguez KA, Espinosa-Arrubarrena L, González-Barba G. An overview of the Mexican fossil fish record. In: Arratia G, Schultze HP, Wilson MVH, editors. Mesozoic Fishes 5 – Global Diversity and Evolution. Verlag Dr. Friedrich Pfeil; 2013. pp. 9–34.

35. González-Barba G, Thies D. Asociaciones faunisticas de condrictios en el Cenozoico de la Peninsula de Baja California, Mexico. Profil. 2000;18: 1–4.

36. Lira-Beltrán RM, González-Barba G, Macías JL, Solis-Añorve A, García-Tenorio F, García-Sánchez L, et al. Fauna de tiburones y rayas de la Formación Tirabuzón (Plioceno) en el Cañón El Álamo, sierras de La Reforma – El Aguajito, Baja California Sur, México. Revista Mexicana de Ciencias Geológicas. 2020;37: 40–63. doi:10.22201/cgeo.20072902e.2020.1.1421

37. Compagno LJV. Galeomorph sharks. In: Parker S, editor. Synopsis and classification of living organisms. McGraw–Hill; 1982. pp. 844–857.

38. Valenciennes A. Les poissons. Troisième. In: Cuvier G, editor. Le règne animal distribué d'après son organisation, pour servir de base à l'histoire naturelle des animaux, et d'introduction à l'anatomie comparée [Troisième] édition..par une réunion de disciples de Cuvier. Troisième. Paris: Fortin, Masson et Co.; 1839. pp. 1–392, pls.

39. Purdy RW. A key to the common genera of Neogene shark teeth. National Museum of Natural History. Smithsonian Institution; 2006.

40. Kent BW. The cartilaginous fishes (chimaeras, sharks, and rays) of Calvert Cliffs, Maryland, USA. In: Godfrey SJ, editor. The Geology and Vertebrate Paleontology of Calvert Cliffs, Maryland, USA Smithsonian Contributions to Paleobiology, 100. Washington, D. C.: Smithsonian Institution Scholarly Press; 2018. pp. 45–157.

41. Betancort JF, Lomoschitz A, Meco J. Los peces (Chondrichthyes, Osteichthyes) del Plioceno inferior de Gran Canaria y Fuerteventura (Islas Canarias, España). Estudios Geológicos. 2016;72: 054. doi:10.3989/egeol.42380.399

42. Ebersole JA, Cicimurri DJ, Stringer GL. Taxonomy and biostratigraphy of the elasmobranchs and bony fishes (Chondrichthyes and Osteichthyes) of the lower-to-middle Eocene (Ypresian to Bartonian) Claiborne Group in Alabama, USA, including an analysis of otoliths. Eur J Taxon. 2019;585: 1–274. doi:10.5852/ejt.2019.585

43. Müller J, Henle FGJ. Systematische Beschreibung der Plagiostomen. Berlin: Veit; 1839. pp 1-200.

44. Poey F. Enumeratio piscium cubensium (Parte III). Anales de la Sociedad Española de Historia Natural. 1876;5: 373–404.

45. Collareta A, Kindlimann R, Baglioni A, Landini W, Sarti G, Altamirano A, et al. Dental Morphology, Palaeoecology and Palaeobiogeographic Significance of a New Species of Requiem Shark (Genus Carcharhinus) from the Lower Miocene of Peru (East Pisco Basin, Chilcatay Formation). J Mar Sci Eng. 2022;10: Article 1466. doi:10.3390/jmse10101466

46. Costa SAF, Richter M, Toledo PM, Moraes-Santos HM. Shark teeth from Pirabas Formation (Lower Miocene), northeastern Amazonia, Brazil. Boletim do Museu Paraense Emílio Goeldi, Ciências Naturais. 2009;4: 221–230.

47. Froese R, D. Pauly. FishBase. World Wide Web electronic publication www.fishbase.org. 2023. 06 June 2023.

48. Pimiento C, González-Barba G, Ehret DJ, Hendy AJW, MacFadden BJ, Jaramillo C. Sharks and rays (Chondrichthyes, Elasmobranchii) from the late Miocene Gatun Formation of Panama. J Paleontol. 2013;87: 755–774. doi:10.1666/12-117

49. Müller J, Henle FGJ. Über die Gattungen der Haifische und Rochen nach einer ... mit Hrn. Henle unternommenen gemeinschaftlichen Arbeit über die Naturgeschichte der Knorpelfische. Bericht über die zur Bekanntmachung geeigneten Verhandlungen der

Königlichen Preussischen Akademie der Wissenschaften zu Berlin. 1837 [ref. 3067];1837: 111–118. Available from: https://www.biodiversitylibrary.org/page/11049813#page/243/mode/1up

50. Dartevelle E, Casier E. Les poissons fossiles du Bas-Congo et des régions voisines. Annales du Musée du Congo Belge, Sér A (Minéralogie Géologie, Paléontologie), 3. 1943;2: 1–200.

51. Agassiz L. Recherches sur les poissons fossiles, 15th and 16th livraisons (March 1843). Jent and Gassmann, Soleure (text) and H Nicolet, Neuchâtel (planches) – vol 3: [i]-[iv], 157-390, 382*-382**, 1–32, [33]-[34], pl 1, 18, 22, 22a, 22b, 26a, 38, 40b, 40c, 40d, 45, 47. 1843.

52. Compagno LJV. Sharks of the order Carcharhiniformes. New Jersey: Princeton University Press; 1988.

53. Argyriou T, Cook TD, Muftah AM, Pavlakis P, Boaz NT, Murray AM. A fish assemblage from an early Miocene horizon from Jabal Zaltan, Libya. Journal of African Earth Sciences. 2015;102: 86–101. doi:10.1016/j.jafrearsci.2014.11.008

54. Andrianavalona TH, Ramihangihajason TN, Rasoamiaramanana A, Ward DJ, Ali JR, Samonds KE. Miocene Shark and Batoid Fauna from Nosy Makamby (Mahajanga Basin, Northwestern Madagascar). PLoS One. 2015;10: e0129444. doi:10.1371/journal.pone.0129444

55. Hasse C. Das natürliche System der Elasmobranchier auf Grundlage des Baues und der Entwicklung ihrer Wirbelsäule. Eine morphologische und paläontologische Studie I Allgemeiner Theil. 1879; 1–76.

56. Agassiz L. Recherches sur les poissons fossiles, 5th livraison (June 1835). Petitpierre et Prince (text) and H Nicolet (plates), Neuchâtel, 1835; vol. 3: pl D, 46.

57. Klunzinger CB. Synopsis der Fische des Rothen Meeres II. Theil Verhandlungen der Königlichen Zoologischen–Botanischen Gesellschaft in Wien. 1871; 441–688.

58. Berg LS. A classification of fish-like vertebrates. Izvestiya Akademii Nauk USSR. Seriya Biologischeskaya. 1937;4: 1277–1280. [In English with Russ. Summ.]

59. Glikman LS. (Sharks of Paleogene and their stratigraphic significance). «in russian». Nauka Press: 1964;Moscou: 29 p., 76 fig., 31 pl.

60. Agassiz L. Recherches sur les poissons fossiles, 11th livraison (November 1838). Petitpierre et Prince (text) and H Nicolet (plates), Neuchâtel, 1838; vol 3: [73]-140, pl 1a, 8a, 8b, 15, 17, 19, 20, 24, 25b, 30, 31, 32, 33, 34, 35, 39, 40..

61. Linné C. Systema Naturae per regna tria naturae, regnum animale, secundum classes, ordines, genera, species, cum characteribus differentiis synonymis, locis. Tomus I. Editio decima, reformata Stockholm, Laurentii Salvii. 1758; 824 pp.

62. Applegate SP, Espinosa-Arrubarrena L. The geology and selachian paleontology of Loma del Tirabuzon (Corkscrew Hill), Santa Rosalia, BCS. Geology of Northwestern Mexico and Arizona Hermosillo: Universidad Nacional Autonoma Mexico, Instituto de Geologia, Estacion de Noroeste. 1981; 257–263.

63. Perez VJ, Godfrey SJ, Kent BW, Weems RE, Nance JR. The transition between Carcharocles chubutensis and Carcharocles megalodon (Otodontidae, Chondrichthyes): lateral cusplet loss through time. J Vertebr Paleontol. 2018;38: e1546732. doi:10.1080/02724634.2018.1546732

64. Alvarado-Ortega J, Cuevas-García M, Cantalice K. The fossil fishes of the archaeological site of Palenque, Chiapas, southeastern Mexico. J Archaeol Sci Rep. 2018;17: 462–476. doi:10.1016/j.jasrep.2017.11.029

65. Berg LS. System der Rezenten und fossilen Fischartigen und Fische. System der Rezenten und fossilen Fischartigen und Fische, Hochschulbücher für Biologie, Berlin. 1958; 310 pp.

66. Müller J, Henle FGJ. On the generic characters of cartilaginous fishes, with descriptions of new genera. Magazine of natural history and journal of zoology, botany, mineralogy, geology and meteorology, (n.s). 1838;2: 33–37; – 88–91.

67. Cappetta H. Les Sélaciens du Crétacé supérieur du Liban. II. Batoïdes. Palaeontographica, Abt A. 1980;168: 149–229.

68. Garman S. New Plagiostomia and Chismopnea. Bull Mus Comp Zool. 1908;51: 249–256.

69. Cappetta H. Les Sélaciens du Miocène de la région de Montpellier. Palaeovertebrata, Mémoire extraordinaire. 1970;1970: 1–139.

70. Geoffroy St. Hilaire E. Poissons du Nil, de la Mer Rouge et de la Méditerranée. In Description de l’Egypte ou recueil des observations et des recherches qui ont été faites en Égypte pendant l'expédition de l’Armée français, publié par les ordres de sa Majesté–L’Empereur Napoléon le Grand. 1817; pl. 18–27.

71. Ebert DA, Stehmann M. Sharks, batoids, and chimaeras of the North Atlantic. FAO Species Catalogue for Fishery Purposes, 7. Rome: Food and Agriculture Organization of the United Nations; 2013.

72. Jordan DS, Evermann BW. The fishes of North and Middle America: a descriptive catalogue of the species of fish-like vertebrates found in the waters of North America, north of the Isthmus of Panama. Part I. Bulletin of the United States National Museum. 1896;47: 1–1240.

73. Cuvier GLCFD. Le règne animal, distribué d’après son organisation, pour servir de base à l’histoire naturelle des animaux et d’introduction à l’anatomie comparée. Edition 2 Paris, Déterville, de l’Impr de A Belin. 1829.

74. Müller J. Untersuchungen €uber die Eingeweide der Fische, Schluss der vergleichenden Anatomie der Myxinoiden. Abhandlungen der Koniglichen Akademie der Wissenschaften zu Berlin. 1845;1843: 109–170. Available from: https://www.biodiversitylibrary.org/page/29197776

75. Betancur-R. R, Broughton RE, Wiley EO, Carpenter K, López JA, Li C, et al. The Tree of Life and a New Classification of Bony Fishes. PLoS Curr. 2013;5. doi:10.1371/currents.tol.53ba26640df0ccaee75bb165c8c26288

76. Bonaparte CL. Prodromus systematis ichthyologiae. Nuovi Annali delle Scienze naturali Bologna (Ser 1) (ann 2) v 4: 181-196, 272-277 [As a separate, p 1-21, distributed 1835! Journal evidently dates to 1840 New taxa date to 1835]. 1835. [ref. 32242]

77. Brisout de Barneville CNF. Note sur les Diodoniens. Revue Zoologique par la Société Cuvierienne (Paris). 1846 [ref. 296]; 136–143.

78. Leis JM. Nomenclature and distribution of the species of the porcupinefish family Diodontidae (Pisces, Teleostei). Mem Mus Vic. 2006;63: 77–90.

79. Nelson JS, Grande TC, Wilson M V. Fishes of the World. John Wiley & Sons; 2016.

80. Böhme M, Ilg A. Fossil fishes, amphibians, reptiles, birds: Localities and taxa from the Permian to the Neogene [Data set]. fosFARbase. http://www.wahre-staerke.com/. 2003.

81. Gallo V, Carvalho MSS de, Souto AA. A possible occurrence of Diodontidae (Teleostei, Tetraodontiformes) in the Upper Cretaceous of the Paraíba Basin, northeastern Brazil. Cretac Res. 2009;30: 599–604. doi:10.1016/j.cretres.2008.12.001

82. Bronn HG. Lethaea geognostica, oder Abbildungen und Berschreigen der für die Gebirgs-Formation bezeichnendsten Verteinrungen. Ed II, Stuttgart. 1855;1–3.

83. Casier E. Deux diodontides Eocenes nouveaux: Progymnodon batalleri nov.sp. et Eodiodon banzai nov. gen. nov. sp. du Bartonien de Belgique. Institut Royal des Sciences Naturelles de Belgique, Bulletin. 1952;28: 1–14.

84. Dames W. Über eine tertiáre Wirbeltierfauna von der westlichen Insel des Birket-el-Querum im fajum (Aegypten). Sitzungsberichte der Königlich Preußischen Akademie der Wissenschaften zu Berlin, Jahrgang 1883 (Stück 6, erster Halbband, Januar bis Mai). 1883; 129–153.

85. Le Danois Y. Sur le remaniement du sous-ordre de poissons Plectognthes et la définition d'un nouveau sous-ordre: les Orbiculates. Comptes Rendus Hebdomadaires de Séances de l'Académie des Sciences (Paris). 1955;240: 1933–1934.

86. Tyler JC, Santini F. Review and reconstructions of the Tetraodontiform fishes from the Eocene of Monte Bolca, Italy, with comments on related tertiary taxa. Studi e Ricerche

sui Giacimenti Terziari di Bolca. Museo Civico di Storia Naturale di Verona. 2002;9: 47–119.

87. Bannikov AF, Tyler JC. First discovery of a porcupinefish (Tetraodontiformes) in the Eocene of the North Caucasus. Paleontological Journal (Moscow). 1997;31: 648–652.

88. Sauvage HE. Mémoire sur la faune ichthyologique de la période tertiaire et plus spécialement sur les poissons fossiles d'Oran (Algérie) et sur ceux découverts par M. R. Alby à Licata en Sicile. Annales des sciences géologiques. 1873;4: 1–272.Taf. 1-18.

89. Dica EP. A review of the Eocene Diodontids and Labrids from Transylvania. Studia UBB Geologia. 2002;47: 37–46.

90. Schultz O. Oligodiodon, ein Igelfisch aus dem Mittel-Miozän (Badenium) der Steiermark, Österreich (Diodontidae, Osteichthyes). Joannea Geol Paläont. 2006;8: 25–46.

91. Santos RS, Travassos S. Contribuição à paleontologia do Estado do Pará . Peixes fósseis da Formação Pirabas. Monografia da Divisão de Geologia e Mineralogia, Departamento Nacional da Produção Mineral. 1960;1960: 1–35.

92. Leidy J. Description of Vertebrate Remains, chiefly from the Phosphate Beds of South Carolina. Journal of the Academy of Natural Sciences of Philadelphia. 1877;2: 209–261, 4.

93. Leriche M. Contribution à l'étude des faunes ichtyologiques marines des terrains tertiaires de la plaine côtière atlantique et du centre des Etats-Unis. Mémoires de la Société géologique de France, Nouvelle Série. 1942;45: 1–111, 8 Taf., Paris.

94. Toula F. Eine jungtertiäre Fauna von Gatun am Panama-Kanal. – Jahrbuch der k.k. Geologischen Reichsanstalt. 1909;58: 673–760, 15 Abb., Taf. 25-28, Wien.

95. Casier E. Contribution à l'étude des poissons fossiles des Antilles. Me m Suisses Paleontol. 1958;74: 1–95, 3 Pl.

96. De Schepper S, Gibbard PL, Salzmann U, Ehlers J. A global synthesis of the marine and terrestrial evidence for glaciation during the Pliocene Epoch. Earth Sci Rev. 2014;135: 83–102. doi:10.1016/j.earscirev.2014.04.003

97. Louys J. Practice and prospects in underwater paleontology. Palaeontologia Electronica. 2018;21.2.22A: 1–14.

Supporting Information captions

S1 File. Video showing the side-mount diving technique used during the dive inside the Cenote San Juan. Underwater recording made with a GoPro Hero 9 black and Bigblue LED video lights.

S2 File. Video showing the sample technique using a chisel and hammer inside the Cenote San Juan. Underwater recording made with a GoPro Hero 9 black and Bigblue LED video lights.

Figure 1

Click here to access/download;Figure;Cantalice_et_al_2024_Plos_Figrue_1.tif

Capa

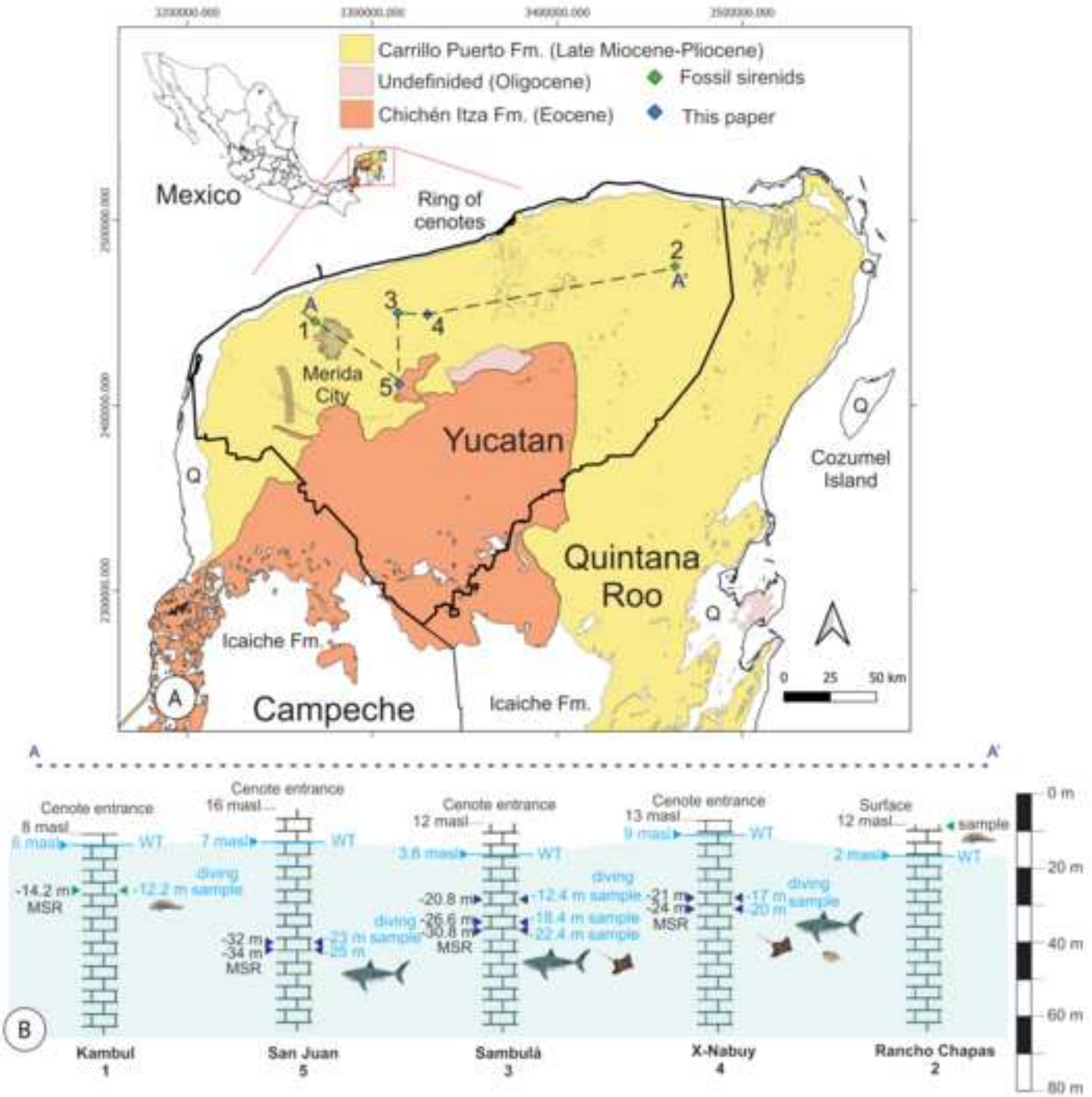

Figure 2

Click here to access/download;Figure;Cantalice_et_al_2024_Plos_Figrue_2.tif

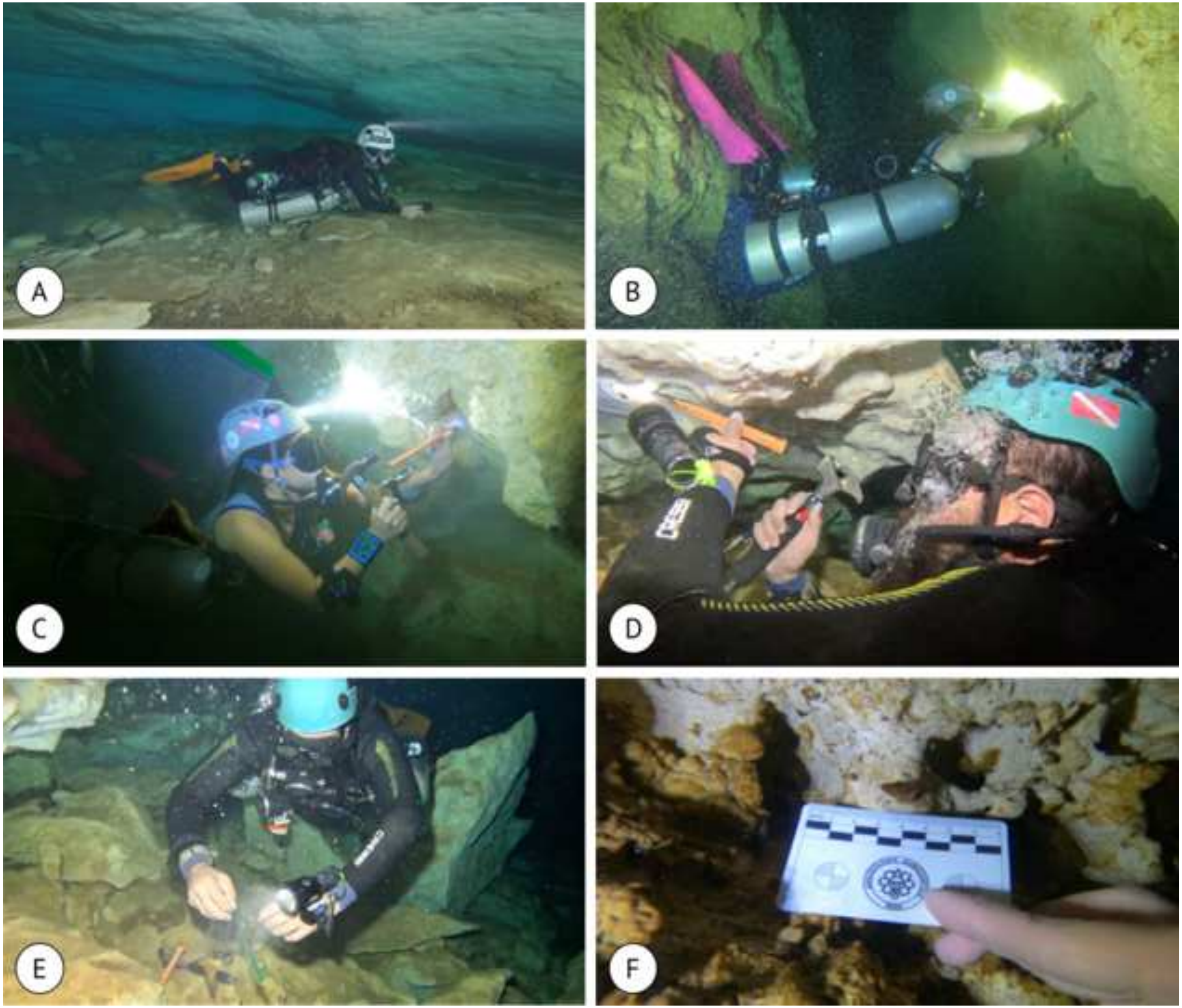

Fig 1.png

Figure 3

Click here to access/download;Figure;Cantalice_et_al_2024_Plos_Figure_3.tif

Brachyurus_final.png

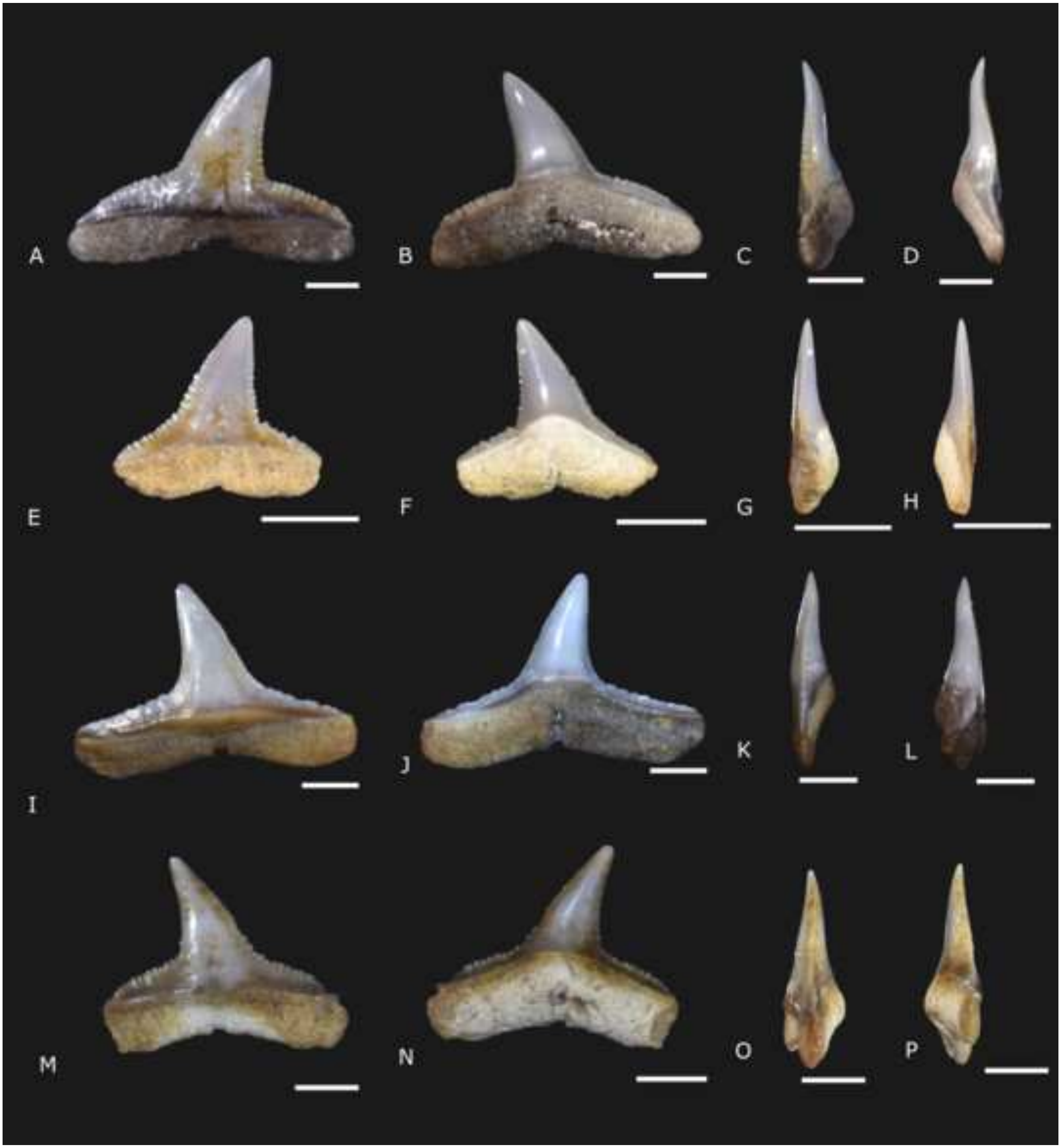

Figure 4

Click here to access/download;Figure;Cantalice_et_al_2024_Plos_Figure_4.tif

Figure_4.tif

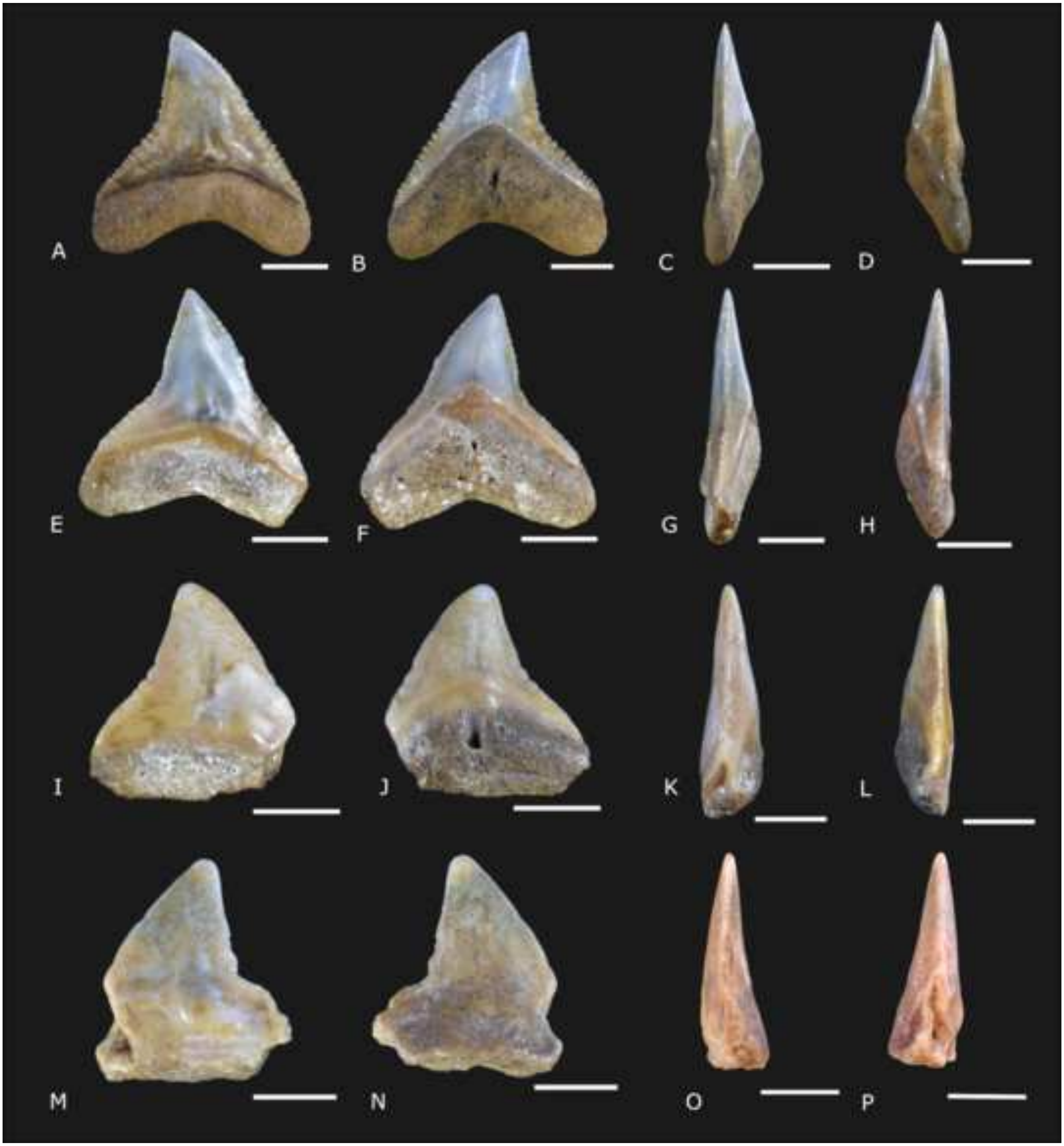

Figure 5

Click here to access/download;Figure;Cantalice_et_al_2024_Plos_Figure_5.tif

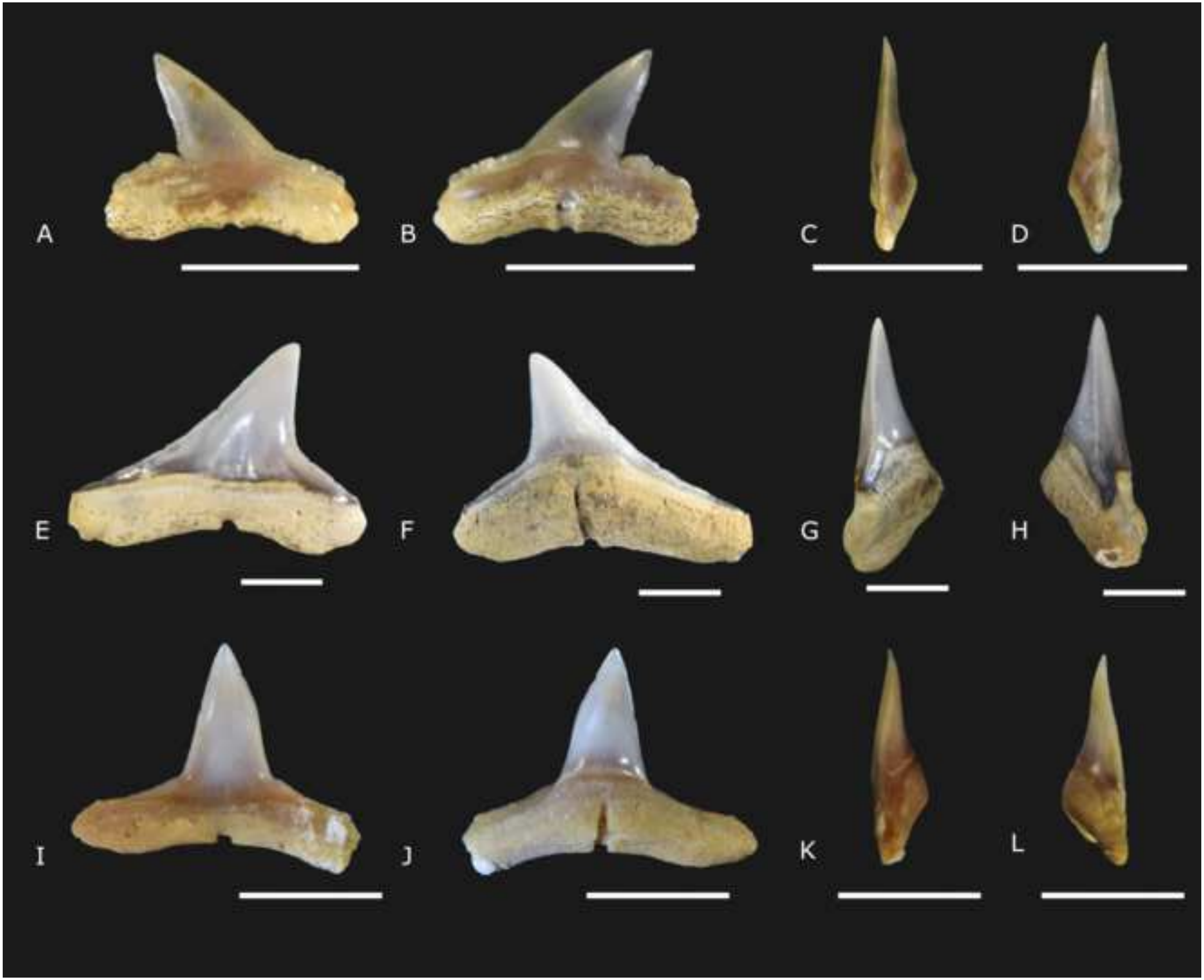

maclot_perezii_sp.png

Figure 6

Click here to access/download;Figure;Cantalice_et_al_2024_Plos_Figure_6.tif

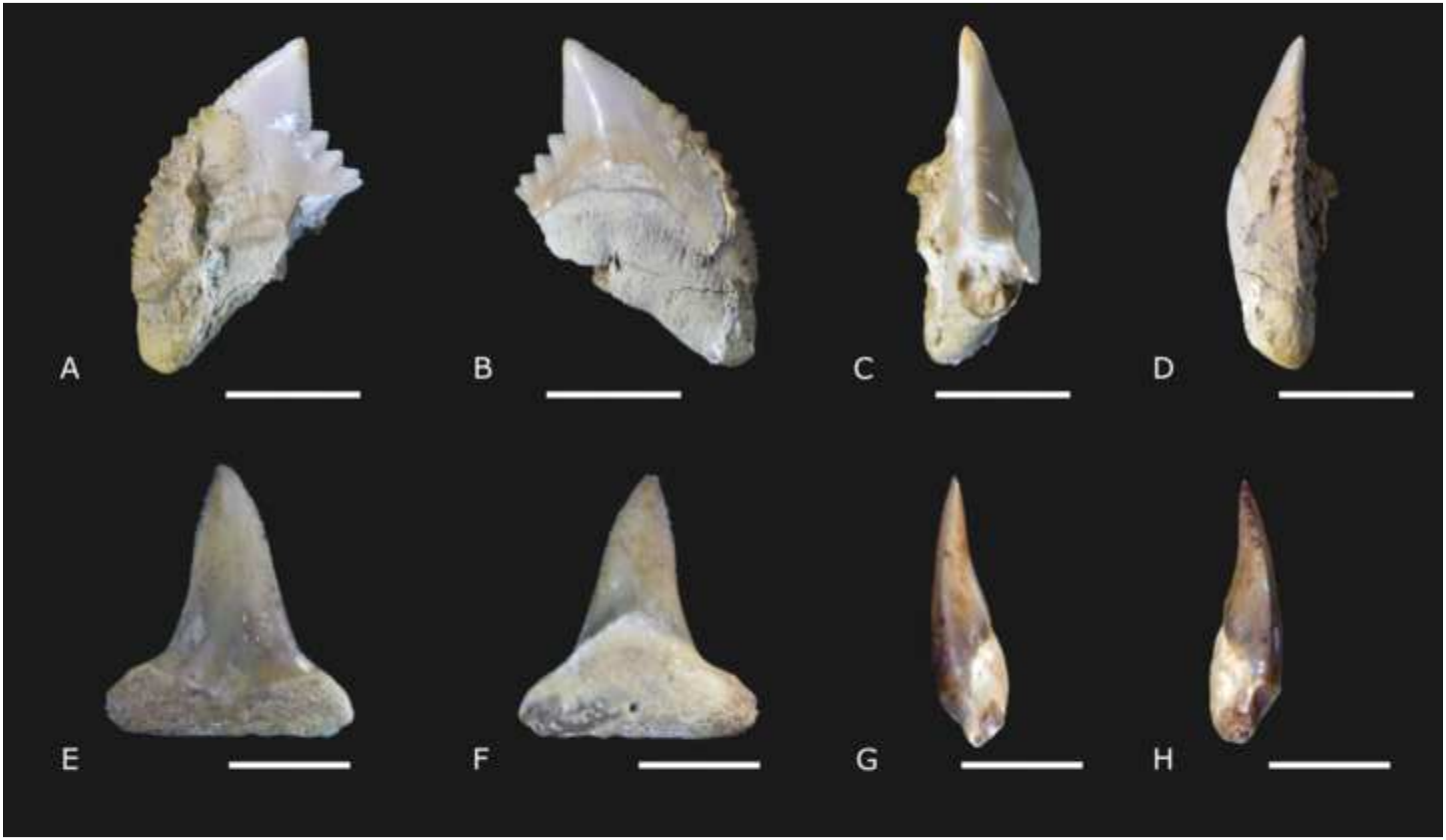

G_Mayumbensis_H_serra_final.png

Figure 7

rect354.png

Click here to access/download;Figure;Cantalice_et_al_2024_Plos_Figure_7.tif

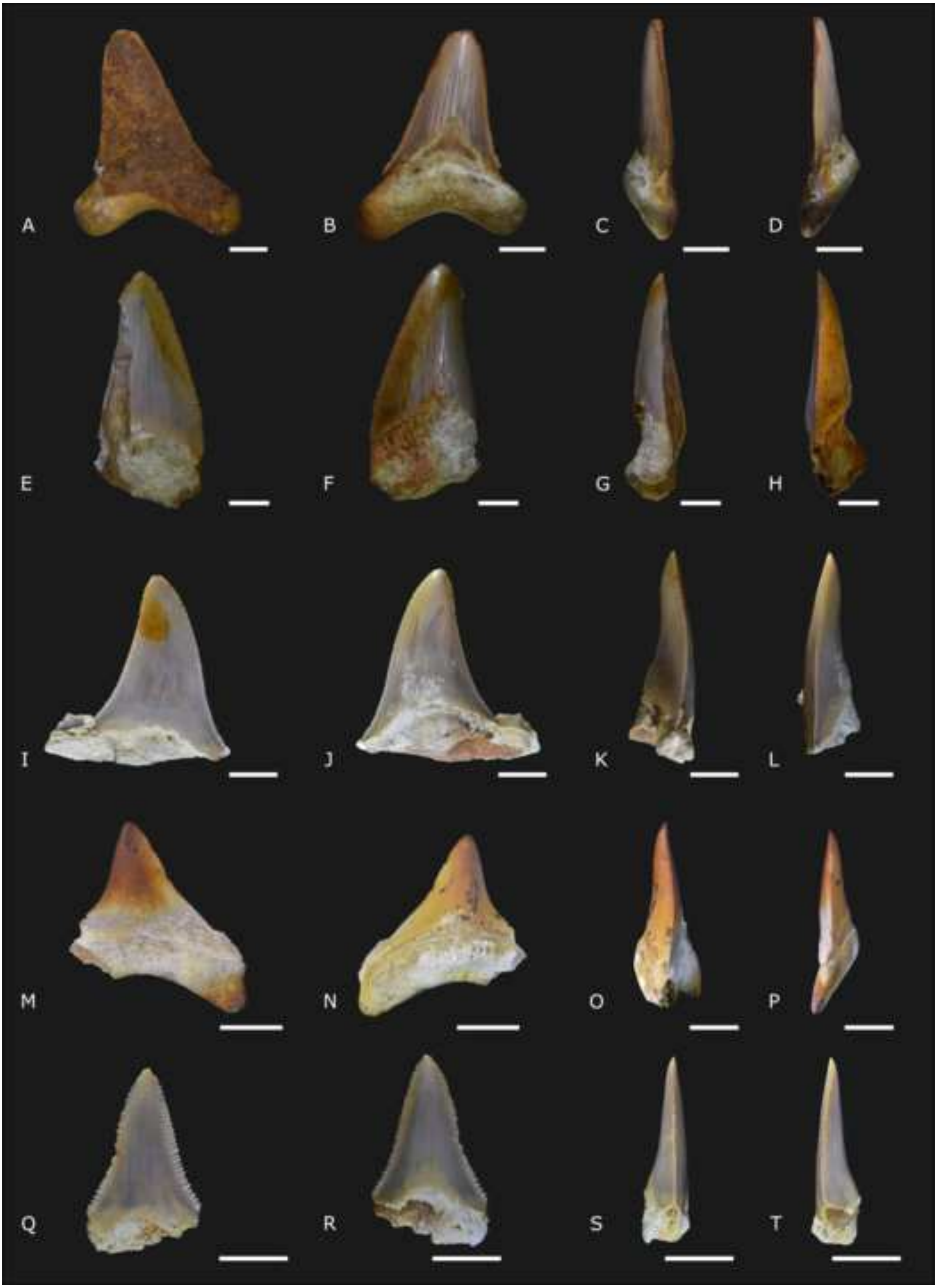

Figure 8

Click here to access/download;Figure;Cantalice_et_al_2024_Plos_Figure_8.tif

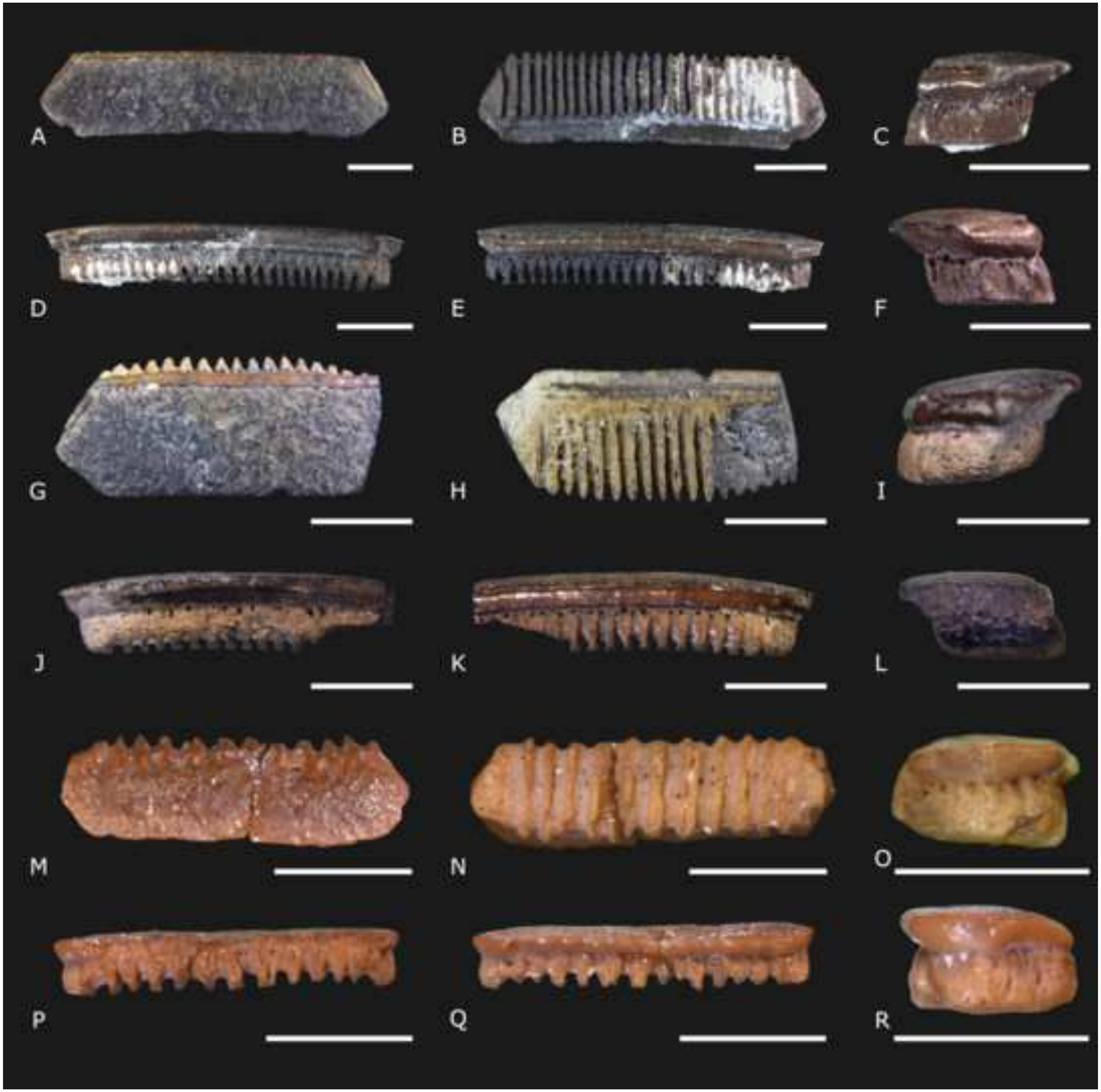

Figure_8.tif

Figure 9

[Click here to access/download;Figure;Cantalice_et_al_2024_Plos_Figure_9.tif]

burr.png

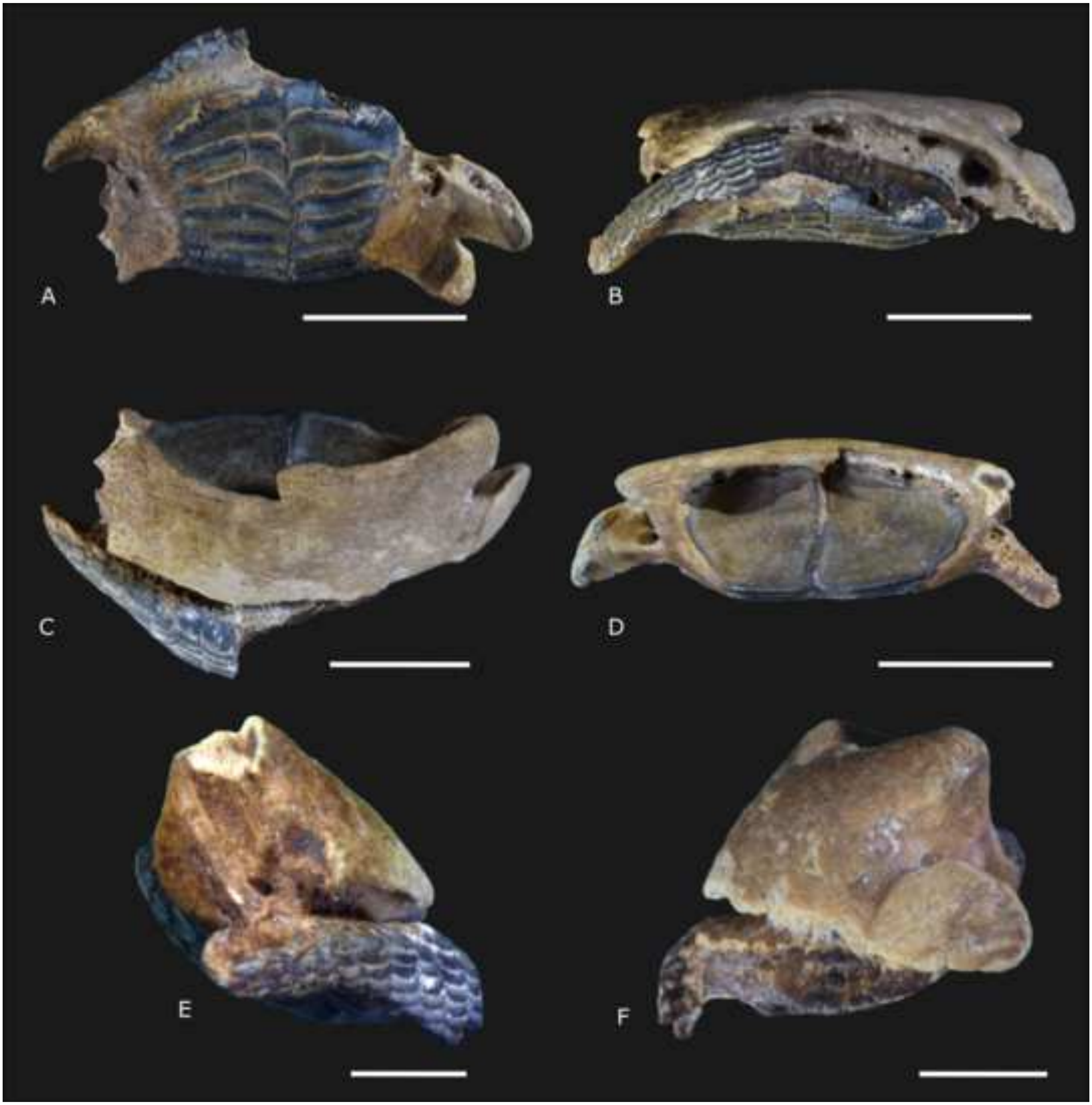

Figure 10

Click here to access/download;Figure;Cantalice_et_al_2024_Plos_Figure_10.tif

Comparison_Int_Tooth_Plate_Burrfish.png

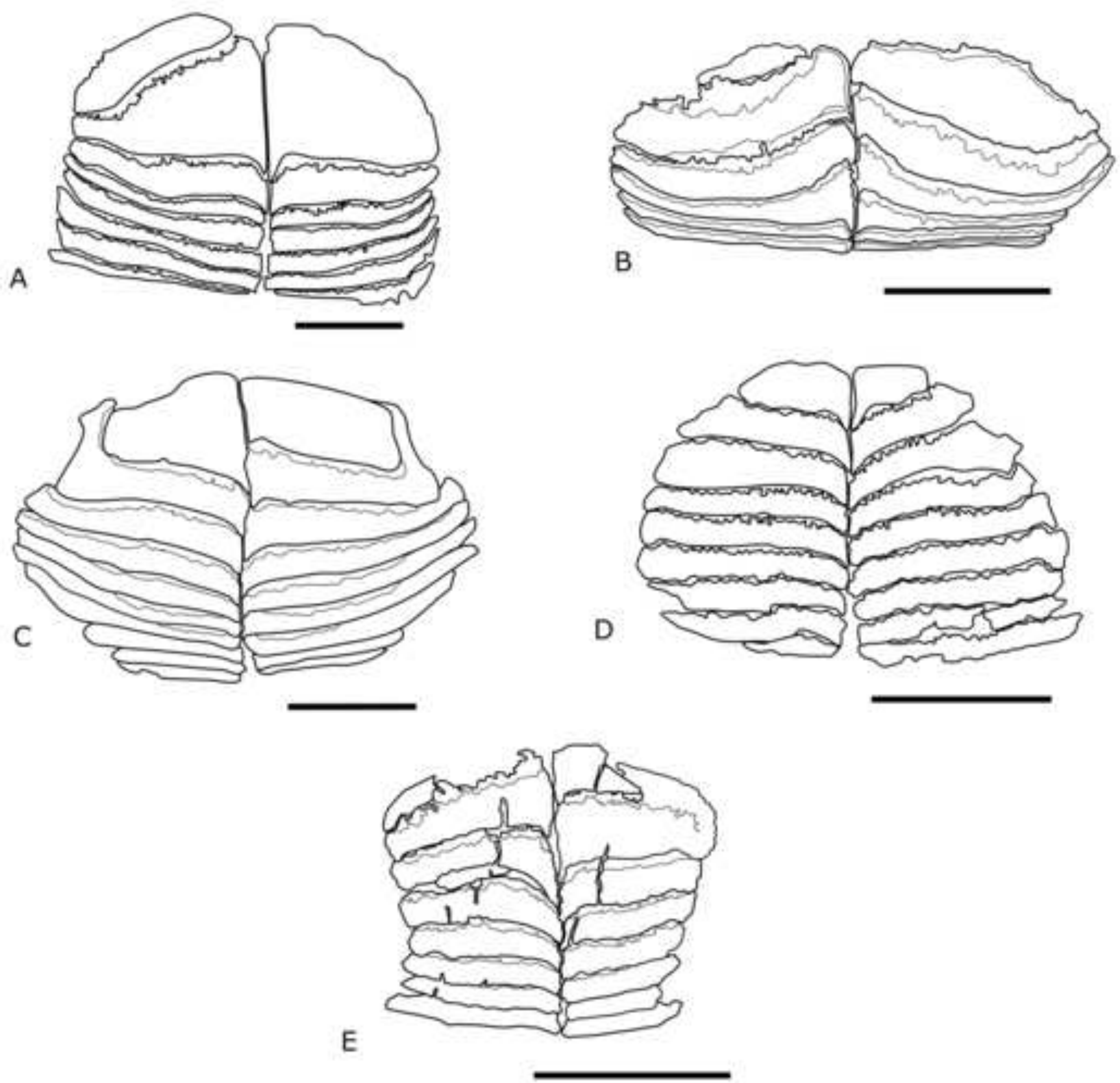

Supporting Information. Video_1_Sidemounting.

Click here to access/download
Supporting Information
Cantalice_et_al_2024_Plos_S1_video_sidemounting.mp4

Supporting Information. Video_2_Collecting.

Click here to access/download

Supporting Information

Cantalice_et_al_2024_Plos_S2_video_colecting.mp4
