# Supplementary material for: Underwater paleontology inside cenotes reveals the Miocene-Pliocene fish diversity in the Yucatan Peninsula, southeast Mexico
Source: PLoS One. 2025 Feb 6;20(2):e0315382. doi: 10.1371/journal.pone.0315382 (PMC11801553; doi:10.1371/journal.pone.0315382)
Supplement: Attachment — Submitted filename: Response to Reviewers.docx [file pone.0315382.s005.docx]

PONE-D-24-07932

Underwater paleontology inside cenotes reveals the Miocene-Pliocene fish diversity in the Yucatan Peninsula, southeast Mexico

PLOS ONE

Response to reviewers

Dear Academic Editor Michael Schubert,

I’m sending this rebuttal letter to address all the points raised during the review process. I want to thank the two reviewers for their comments and suggestions, which were fundamental to improving the final version of the manuscript.

Sincerely,

Kleyton M. Cantalice

**Response to journal requirements:**

When submitting your revision, we need you to address these additional requirements.

1. Please ensure that your manuscript meets PLOS ONE's style requirements, including those for file naming. The PLOS ONE style templates can be found at

https://journals.plos.org/plosone/s/file?id=wjVg/PLOSOne_formatting_sample_main_body.pdf and

<https://journals.plos.org/plosone/s/file?id=ba62/PLOSOne_formatting_sample_title_authors_affiliations.pdf>

**Response.** The file naming for the figures and the supplementary information were modified. The table style was also modified to meet PLOS ONE’s style requirements.

2. Manuscripts reporting paleontology and archaeology research must adhere to our policies described at http://journals.plos.org/plosone/s/submission-guidelines#loc-paleontology-and-archaeology-research. Specifically, appropriate identification numbers for the human remains, specimens and/or samples should be provided, and the data used in the study should be publicly deposited or made accessible for replication of the study. If applicable, please ensure permission to conduct destructive sampling was obtained. Only proceed to acceptance after these requirements are met.

**Response.** Appropriate identification numbers were provided. The data are housed in a public collection and are accessible for study replication. Permission to conduct destructive sampling was not applicable.

In keeping with PLOS ONE’s standard policies, please check that you have not published with any of the authors of this submission within the last 5 years. The journal asks Academic Editors to recuse themselves from handling a manuscript if they have a potential competing interest, including recent co-publications. For more information on PLOS ONE’s competing interests policy for Editors see here: <https://journals.plos.org/plosone/s/competing-interests#loc-who-must-declare-competing-interests>.

**Response.** I have not published with any of the authors of this submission within the last 5 years.

3. Thank you for stating the following financial disclosure:

“KMC. Grant Number: IA206123. Dirección Genral de Asuntos del Personal Académico (DGAPA) - Programa de Apoyo a Proyectos de Investigación e Innovación Tecnológica (PAPIIT).

Please state what role the funders took in the study.  If the funders had no role, please state: "The funders had no role in study design, data collection and analysis, decision to publish, or preparation of the manuscript."

**Response.** The role of the funder in the study includes specimen collection and data analysis.

If this statement is not correct you must amend it as needed.

Please include this amended Role of Funder statement in your cover letter; we will change the online submission form on your behalf.

**Response.** This information was included in the cover letter.

4. When completing the data availability statement of the submission form, you indicated that you will make your data available on acceptance. We strongly recommend all authors decide on a data sharing plan before acceptance, as the process can be lengthy and hold up publication timelines. Please note that, though access restrictions are acceptable now, your entire data will need to be made freely accessible if your manuscript is accepted for publication. This policy applies to all data except where public deposition would breach compliance with the protocol approved by your research ethics board. If you are unable to adhere to our open data policy, please kindly revise your statement to explain your reasoning and we will seek the editor's input on an exemption. Please be assured that, once you have provided your new statement, the assessment of your exemption will not hold up the peer review process.

**Response.** No access restrictions are needed.

5. One of the noted authors is a group or consortium [Underwater Archaeological Atlas project^]. In addition to naming the author group, please list the individual authors and affiliations within this group in the acknowledgments section of your manuscript. Please also indicate clearly a lead author for this group along with a contact email address.

**Response.** Helena Barba Meinecke coordinates the Underwater Archaeological Atlas Project. We aggregate the information required in the acknowledgments section of the manuscript. If the contact email is in another place, please let me know and make the appropriate modifications

6. We note that Figure 1 in your submission contain [map/satellite] images which may be copyrighted. All PLOS content is published under the Creative Commons Attribution License (CC BY 4.0), which means that the manuscript, images, and Supporting Information files will be freely available online, and any third party is permitted to access, download, copy, distribute, and use these materials in any way, even commercially, with proper attribution. For these reasons, we cannot publish previously copyrighted maps or satellite images created using proprietary data, such as Google software (Google Maps, Street View, and Earth). For more information, see our copyright guidelines: http://journals.plos.org/plosone/s/licenses-and-copyright.

**Response.** The authors made Figure 1 of the work, which has never been published. The spatial data of the geological formations were KML data files obtained from the Mexican Geological Service (SGM), whose information corresponds to government open data with its due acknowledgment of authorship, i.e., citation. The credit is included in the figure caption and references, following the steps requested by the SGM for authorization.

The steps for doing Figure 1 were as follows: 1. First, we downloaded KML data 2. After obtaining the data, the image was processed in QGIS and Corel Draw software.

The geospatial data are available for download at the following link https://www.sgm.gob.mx/GeoInfoMexGobMx/

https://datos.gob.mx/busca/dataset/cartografia-geologica-de-la-republica-mexicana-escala-1-250000

Government free-use statement

https://datos.gob.mx/libreusomx

7. Please review your reference list to ensure that it is complete and correct. If you have cited papers that have been retracted, please include the rationale for doing so in the manuscript text, or remove these references and replace them with relevant current references. Any changes to the reference list should be mentioned in the rebuttal letter that accompanies your revised manuscript. If you need to cite a retracted article, indicate the article’s retracted status in the References list and also include a citation and full reference for the retraction notice.

**Response.** The references [4-10] and [90] were removed. The reference numbers were actualized. The old reference [73] was moved to the new [62] to follow the text's appearance order. Small modifications (italic) in the new references [9-10].

[Note: HTML markup is below. Please do not edit.]

Reviewers' comments:

Reviewer's Responses to Questions

**Comments to the Author**

1. Is the manuscript technically sound, and do the data support the conclusions?

The manuscript must describe a technically sound piece of scientific research with data that supports the conclusions. Experiments must have been conducted rigorously, with appropriate controls, replication, and sample sizes. The conclusions must be drawn appropriately based on the data presented.

Reviewer #1: Yes

Reviewer #2: Yes

2. Has the statistical analysis been performed appropriately and rigorously?

Reviewer #1: N/A

Reviewer #2: N/A

3. Have the authors made all data underlying the findings in their manuscript fully available?

The [PLOS Data policy](http://www.plosone.org/static/policies.action#sharing) requires authors to make all data underlying the findings described in their manuscript fully available without restriction, with rare exception (please refer to the Data Availability Statement in the manuscript PDF file). The data should be provided as part of the manuscript or its supporting information, or deposited to a public repository. For example, in addition to summary statistics, the data points behind means, medians and variance measures should be available. If there are restrictions on publicly sharing data—e.g. participant privacy or use of data from a third party—those must be specified.

Reviewer #1: Yes

Reviewer #2: No

4. Is the manuscript presented in an intelligible fashion and written in standard English?

PLOS ONE does not copyedit accepted manuscripts, so the language in submitted articles must be clear, correct, and unambiguous. Any typographical or grammatical errors should be corrected at revision, so please note any specific errors here.

Reviewer #1: Yes

Reviewer #2: No

5. Review Comments to the Author

Please use the space provided to explain your answers to the questions above. You may also include additional comments for the author, including concerns about dual publication, research ethics, or publication ethics. (Please upload your review as an attachment if it exceeds 20,000 characters)

Reviewer #1: This manuscript presents a species inventory for a new Mio-Pliocene locality in the Yucatan Peninsula, expanding our understanding of the shark and teleost diversity. Of particular interest is that the locality is only accessible diving.

Results indicate that the vertebrate fauna recovered from this locality is consistent with invertebrate faunas, which predict an shallow shelf neritic zone of deposition. This work further expands the taxonomic diversity of the formation. It also points out that these specimens recovered are disproportionately smaller than from other Mio-Pliocene localities. This lead authors to hypothesize that the locality was a shelter for sub-adult individuals (aka, a nursery).

As a reader, I'd like to know more about the evidence supporting the sub-adult nursery hypothesis. It is among the most interesting aspects of the paper and should come earlier in the discussion. The species inventory and table 1 can be used to support that all specimens and taxa are in fact small. Then go into why all of these might be small due to ontogeny vs some other size-variability considerations. You currently have one example for Otodus megeladon, but what else?

**Response.** We include the nursery hypothesis later in the text because we decided to consider taxonomic aspects first. The megalodon teeth are a clear example of a sub-adult species because of the secondary cusplets. Nevertheless, the small size is a feature of all teeth specimens in the Carrillo Puerto Formation.

In addition, the second most interesting thing is the new taxon of burrfish. This warrants more time in the discussion. To some extent, I'd consider moving some of what is listed in remarks (pages 34-37) to the actual discussion section for the entire paper, to get the most readership.

**Response.** Suggestion accepted.

Other structural considerations:
The use of Table 1 sooner may help you organize the Systematic Paleontology descriptions. Then you can reduce the descriptions to only the new ones from this study - while validating why it isn't something that was previously published by Domning (1989/1990).

**Response.** Domning (1989, 1990) just mentioned the possible presence of some species we described here. Although previously mentioned, this is the first time fish species from Carrillo Puerto have been formally reported, including catalog number, diagnosis features, etc. Therefore, we believe that all descriptions must remain in the manuscript.

Taxonomy - I'm most familiar with batoids and teleosts, thus a bit of a heavy discussion on specimens in figure 8.
Pteromylaeus is a synonym of Aetomylaeus (Garman 1908). The description and morphology of that specimen is consistent with it's placement in Aetomylaeus as well. Incredibly fine tooth roots, as narrow as the space between roots, step like displacement of roots in the labial direction. No interlocking ridge and grove as in rhinobatos. For the sharks teeth, I will need to defer to another reviewer to confirm synonymy and correct placement.

**Response.** Changed. *Pteromylaeus* to *Aetomylaeus*. Thank you.

Discussion - The discussion is a little underwhelming. There is too much review of what was previously known and the significance of the fish biodiversity comes late. See notes above. I want to read more about the impact of these fossils and about the locality.

**Response.** We believe the discussion is the moment to compare our results with the previously published works. Because of this, we put the information about biodiversity first and the contribution of our study afterward. For example, the first paragraph in the fossil diversity section of the Carrillo Puerto Formation talks about the formally described species. The second concerns the not formally described ones and our contribution to understanding the Carrillo Puerto fossil elasmobranch diversity. The last paragraph is a small discussion about the new diodontid species described.

After this section, the discussion continues with a detailed discussion of the diodontid family (such as suggested by the reviewer) and an explanation of the diagnostic features that allow us to determine this fish as a new diodontid species belonging to the genus *Chilomycterus*. The information about the impact of these fossils and the locality was in the Fish diversity significance section of the Discussion. Our study aims to provide the first formal description of the fossil fish biodiversity from the Carrillo Puerto Formation. This study is essential because this first fossil fish inventory supports determining that the fish diversity is more significant than previously thought. The small-sized teeth observed (together with sub-adult features found in megalodon teeth) suggest that this locality probably was a nursery. Unfortunately, the small number of well-preserved specimens does not allow us to perform a more detailed statistical study to give support for our hypothesis about the past environment in the Carillo Puerto Formation. We hope to increase the number of specimens posteriorly.

Figures - At minimum for Figure 9 of the new taxon, you need to add labels and point out any apomorphic trait. Ideally, you will do the same for the other teeth as well.

**Response.** The labels were added.

Specific Line Comments:
page 3, line 74. Change "evidence the presence" to "preserve"
page 30, line 643. Indicate what anterior and posterior.
page 31, line 659, add the word burrfish before the word specimens.
page 31, lines 658-661. This is a long sentence that can be broken up for clarity.
page 31, line 662. "last pair" - is this anterior or posterior?
page 32, line 673, change "opposite" to "antemere"
page 32, line 687, change "teeth' vertical series" to "vertical series of teeth"
page 32, line 690, add a comma after beak
page 37, line 791. Porrly known because... Add more information.

**Response.** The detailed information about the Carrillo Puerto follows this sentence

page 38, table 1: be consistent in the placement of the term "this study" so that it is always first before another reference
page 39, line 805: Change first word, "On" to "Prior work on"
page 39, line 817. change "presents" to "comprises"
page 39, line 821. The word "exposed" - this comes up in a few places and needs a better definition and label associated with illustrations/figures. Describe wgat that actually means and how you know that it is real morphology vs taphonomy.

**Response.** An explanation of our conclusion that this feature is a natural morphological feature and not taphonomy was included in the description section. At the end of the fourth paragraph.

page 41, line 853, delete the word 'interesting'

page 41, line 861. What does "posterior extinction" mean?

**Response.** We are trying to explain that the megalodon was extinguished posteriorly. The sentence was removed.

page 42, lines 878-883. Awkward sentence structure.

**Response.** The sentence was changed.

Reviewer #2: The authors describe the fish fossil assemblage recovered from the Miocene-Pliocene deposits of the Carrillo Puerto Formation located in the Yucatan Peninsula, Mexico. The analyzed specimens include shark taxa from the Mexican coast and a newly identified diodontid fish. The assemblage, along with the environmental interpretation of the deposits, suggests that these taxa inhabited a shallow water habitat, possibly serving as a nursery area.
I thoroughly enjoyed this paper and think this manuscript deserves to be published on Plos One, particularly for the sampling method. It blew my mind when I realized that you scuba-dived in caves to recover the specimens — an incredibly challenging and impressive feat. I here recommend this manuscript for publication in Plos One after minor revisions given the following general comments:

1) Use standard terminology to describe shark teeth and guide readers through each morphological and morphometric feature. Please include a schematic figure in the methods section to explain these traits. Additionally, the proportions you reported do not sum to 100%, which raises concerns about your measurement methods. Lastly, I have never seen a shark tooth dimension described as “depth.” Do you mean height? Please reword this.
**Response.** The terminology was standardized. The schematic figure was included. The proportions are more than 100% because of the nature of shark teeth. The schematic figure explains why these proportions exist. All the manuscript was reworded with the word height. Thank you.

2) You cannot definitively state that the Carrillo Puerto Formation was a nursery area without quantitative analysis to support this hypothesis. Include all measurements in the supplementary materials and reference this table to substantiate your interpretation. If you aim to corroborate this hypothesis, you must include body size estimates and the range of body sizes for modern analogs at different ontogenetic stages for comparison. Otherwise, stay general.
**Response.** The table was performed and included as supplementary data.

3) The main text needs polishing. Some words are misspelled, while others seem out of context. I have included suggestions to reword some paragraphs, but I recommend carefully reviewing the manuscript before the resubmission.
**Response.** The manuscript was revised, and the text was polished.

These are general comments, with more specific ones provided in the main text. Good luck with the revision and I look forward to seeing your manuscript published on Plos One.

**Main text responses:**

Lines 33-35. Changed to “hosts a unique underwater karstic system made of galleries connected by multiple sinkholes, locally called cenotes.”

Lines 37-38. Changed to "of these deposits and expose the fossil assemblage."

Line 42. Changed to “extinct taxa.”

Lines 44-45. Changed to “taxa that are not currently distributed in Mexican coasts, such as *Carcharhinus macloti* and representatives of the genus *Pteromylaeus*.”

Line 57. Removed the word “solutions” and changed “sinkhole” to “sinkholes”.

Line 59. Added the word “Mexico”.

Lines 63-67. Sentence removed.

Lines 68-69. Sentence removed.

Lines 69-70. Removed “Earth science research indicates that”

Line 73. Changed “A great portion” to “Most”

Line 73. Removed “is formed by coquina strata, which”.

Lines 79-80. Moved to line 59.

Line 79. Changed “prospections” to “sampling”.

Lines 83-88. Changed to “The Carrillo Puerto Formation outcrops in the Yucatan Peninsula over an area of about 8800 Km2, which includes the central and eastern part of the state of Quintana Roo, the central region of Yucatán, and northeastern Campeche. It comprises limestones that classified into…”

Line 87. Changed “five” to “5”.

Line 96. Changed “masl” to “m.a.s.l.”.

Line 104. Removed the word “impure”.

Line 114. Changed “Formations” to “formations”.

Line 114. Added the word “Mexico” after “Veracruz State”.

Line 117. Changed “it is considered a shallow shelf depositional environment with internal neritic conditions and middle neritic influence” to “the environment was interpreted as a shallow marine habitat corresponding to the neritic zone”.

Line 119. Changed “correspond to a reasonably” to “persisted for a prolonged period”

Line 148. Removed the italic font and the translation. However, we prefer to keep the Colección Nacional de Paleontología since it is the specific place in the IGM to accommodate the fossil specimens.

Lines 149-150. Removed italic font and translation.

Line 164. Changed “Filo” to “Phylum”.

Lines 177-178. Changed “Right lateral” to “Distal” and “Left lateral” to “Mesial”.

Lines 188-190. Changed “depth” to “height”.

Line 192. Changed “lateral” to “in both medial and distal”. The mentioned features can be viewed in both views.

Line 207. Changed “Copper” to “copper”.

Lines 214-216. Changed “Extant *C. brachyurus* reaches up to 320 m in marine environments and is generally associated with inshore and reef systems; however, it is reported in estuaries and occasionally in freshwater [37]“ to “Extant *C. brachyurus* reaches up to 320 m in marine environments and is associated with reef systems, estuaries, and occasionally in freshwater [37]”.

Line 237-238. Added “to the distal edge”

Line 243. Changed “Cusplet” to “Cusplets”

Line 252. Changed “Bull” to “bull”.

Lines 260-262. Removed “;however”, and added “,and”

Line 274. Added “(Fig 5 A-D)”.

Line 278. Reference figured.

Line 281. Every inclined tooth is pointed distally.

Line 282. Changed “;” to “,”.

Lines 282-283. Changed “lingual is medially concave” to “while the lingual surface is medially concave, forming a sigmoid curvature observed in both distal and mesial views.”

Line 285. Changed “about” to “around”

Line 302. Changed “Hardnose” to “hardnose”

Lines 306-307. Changed “the lack of the twist in the crow” to “absence of twist in the crown”

Line 307. Added “found in”.

Line 321. The crown can be upright or curved. The curvature can be to the right or the left side. In all curved crowns, the curvature is directed to the distal portion. In this case we are indicating the direction of the crown curvature.

Line 332. Removed “in all extensions”.

Line 336. Removed “forming a blade that also is finely serrated.”

Lines 343-346. The sencentes was changed to “This single specimen is attributed to C. perezii (the Caribbean reef shark) due to the presence of diagnostic features, including a large and triangular crown with vestigial serrations along both the cutting edges and the curvature of the cutting edges not pronounced”.

Line 352. Removed italic from sp.

Line 358. Fig 1 referenced.

Line 361. Changed “unique” to “single”.

Line 361. Changed “depth” to “width”.

Lines 366-368. Sentence changed.

Lines 369-371. Changed “degrees” to °. Changed along the main text.

Line 373. Changed to “The shoulders are wide and the enameled portion over its dorsal surface is thin”.

Line 382. Changed Requiem to requiem.

Line 383. Changed shoulder to shoulders.

Line 384. Changed to and a notable transverse groove.

Line 404. Added (Fig 1).

Line 407. Changed unique to single.

Lines 411-412. Rewrite as “In both mesial and distal views, the labial surface of the crown is concave, while the lingual is convex.”

Lines 412. Sentences changed to: The apex in *Galeocerdo mayumbiensis* is directed to the labial portion but does not have the apex sigmoid curvature, such as is found in Carcharhinus sp. for example.

Lines 421-423. Paragraph changed.

Lines 427-432. Paragraph changed.

Line 437. Changed

Line 439. Word removed.

Line 450. Word removed.

Line 454. Changed unique to single

Line 455. Changed to wider than long.

Line 462 Changed to snaggletooth

Line 462. Removed the word jaw.

Line 495. Added (Fig 1).

Line 499. Changed depth to width.

Line 501. Changed crow to crown.

Line 502. Added In both mesial and distal views.

Line 507. Removed the word shallower.

Line 520. The sentence was changed to “teeth with the crown labio-lingually flattened”.

Line 521. Changed to well-developed.

Line 524. The sentence was changed to “The small length indicates that all teeth belonged to small specimens, probably juvenile sharks.”

Line 527. Added: of †*Otodus (Carcharocles) megalodon.*

Line 537. Added: (Fig 7 Q-T).

Line 544. Changed to: The single tooth is flattened...

Line 546. Added: in both mesial and distal views.

Line 548. Changed to “strongly serrate”

Line 555. Changed to The single specimen.

Line 555. Changed to white

Line 558-560. Sentence changed to: Some hypotheses indicate that these species are not contemporary [39]. Nevertheless, a previous co-occurrence reported in the late Pliocene strata of Baja California [62] and the single species herein described indicates that both species occurred on both sides of the Mexican coasts during the Miocene-Pliocene.

Line 578. Changed almost to nearly.

Line 581. Changed the word X-Nubay to X-Nabuy, and figure 1 was referenced.

Line 584. Removed slightly tacky.

Line 587. Schematic drawing

Line 588. Schematic drawing

Line 589. Schematic drawing

Line 591. Schematic drawing

Line 594. Changed to “are classified under”

Line 595. Changed to well-developed foramina.

Line 597. Changed to the taxonomic rank at the species level.

Line 610. Changed to: a small and incomplete tooth plate, with light brown coloration.

Line 611. Added (Fig 1).

Line 625. The paragraph was reduced.

Line 625. Changed to the singles specimen

Line 632. Sentence removed.

Line 653. Added (Fig 1)

Line 655. LSID means Life Sciences Identifiers, an identifier for Zoobank registration. It will be available after the acceptance of the manuscript.

Lines 658 to 661. Paragraph divided.

Line 663. New table table.

Line 668. Changed to latinization.

Lines 668-669. The sentence was changed to which the Mayan people used to refer to the sinkholes

Line 670. Changed to that.

Line 672. Schematic drawing.

Line 678. Sentence removed.

Line 687. Changed to vertical series of teeth.

Line 690. Changed to scrap the food. Reference added.

Line 697. Removed the word the

Lines 697-698. Sentence changed to In ventral view, it is possible to observe the external surface of the trituration plate of the upper jaw.

Line 698. Changed Space to Gap

Line 714. Changed to fish ad burrfish

Line 725. Added specimen

Line 734. Changed genus to taxon

Line 738. Semicolon removed

Lines 740-741. Added the sentence contrasting with †C. dzonotensis, that have the plates unparallel with a parabolic increase.

Lines 742-743. Sentence changed to During the Neogene, the number of diodontid genera was reduced, however, it was the moment when species of extant genera rose.

Line 746. Semicolon removed.

Line 751. The species names are in the legend of the figure.

Line 781. Changed possess to have a.

Lines 782-783. Added a diagnostic feature of Chilomycterus specimen.

Line 786-787. Changed the sentences to †C. dzonotensis is the first diodontid fossil from the Miocene-Pliocene strata of the Gulf of Mexico.

Line 798. Changed to vertebral remains.

Line 809. Sentence changed to, and two tiger sharks: one related to †Galeocerdo rosaliensis Applegate, 1978, and the other with †G. aduncus [17].

Line 810. Changed author to Domning [16,17].

Line 817. Changed recognize to classified.

Line 818. semicolon removed.

Line 820. Semicolon removed.

Lines 828-829. The figure 1 was referenced.

Line 837-838. The sentence has been changed to: Remarkably, the remains of chondrichthyans and teleost fossil fish in sinkholes from the Carrillo Puerto Formation are small compared with other fossil and extant adult species.

Lines 841-842. Rewrite making a comparison

Line 843. Changed was to could be.

Lines 853-855. The sentence has been changed to: Another point to consider with the fish fauna from the Carrillo Puerto Formation is the repeated co-occurrence of the white shark and megalodon on Mexican coasts during the Miocene-Pliocene.

Line 854. Changed to white shark and megalodon

Line 860. Changed to white and megalodon

Line 871. Semicolon removed.

Lines 872-873. Other taxa were included in the discussion. Such as *Pteromylaeus* and *C. macloti*

Lines 879-883. Semicolons removed. Sentence changed.

Line 891. Changed corroborates to supports.
