# Supplementary material for: Underwater paleontology inside cenotes reveals the Miocene-Pliocene fish diversity in the Yucatan Peninsula, southeast Mexico
Source: PLoS One. 2025 Feb 6;20(2):e0315382. doi: 10.1371/journal.pone.0315382 (PMC11801553; doi:10.1371/journal.pone.0315382)
Supplement: Attachment — Submitted filename: Response to reviews.docx [file pone.0315382.s006.docx]

Response to reviews.

Dear Editor in Chief and reviewer,

Here is a rebuttal letter responding to each point raised. All the journal requirements were attended to, and some modifications were made to the references (in the manuscript and the reference section) to accommodate the journal standards. All the modifications in the references are mentioned below.

The modifications proposed by the reviewer were also attended to. The hypothesis about the nursery and the presence of juveniles were completely removed, and the manuscript focused only on saying that some specimens in the Carrillo Puerto Formation were small.

Some other small corrections to the manuscript and table S1 were performed.

Thank you so much for the observations and suggestions for improving the final version of the manuscript.

Sincerely,

Kleyon M. Cantalice

Below are the modifications performed in the manuscript and the table:

Line 46. Changed the word “all” for “some.”

Line 68. Reference 6 added.

Line 83. Changed SGM to Servicio Geológico Mexicano (SGM). It is a free Access document provided by the Geological Service of Mexico, which has no authors nor cited date.

Line 104. Reference 12 added.

Line 215. Reference 25 added.

Line 217. Reference 28 added.

Line 260. Reference 34 added.

Line 326. Changed the word “in” to “on”.

Line 384. Changed “1843” to “1838”, and “[44]” to “[53]”. I found a mistake in the citation of the species †*Hemipristis serra* in Figure 7 caption, and †Otodus (Carcharocles) megalodon in Figure 8. Because of this modification, the reference [44] was removed, and the number of the other references was accommodated.

Line 418. Changed [45] to [44], and [46] to [45].

Line 421.Changed [46] to [45], and [47] to [46].

Line 428. Changed 48 to 47.

Line 429. Changed 49 to 48.

Line 431. Changed 49 to 48.

Line 454. Changed 47 to 46.

Line 460. Changed 51 to 50.

Line 461. Changed 52 to 51.

Line 462. Changed 53 to 52.

Line 464. Changed 1843 to 1838, and 53 to 52.

Line 467. Changed 1843 to 1838, 44 to 52.

Line 474. Changed “the root” to “root”.

Line 484. Changed “height” to “height ranges”.

Line 495. Changed “separate” to “separated”.

Line 497. Changed “lesser” to “less”.

Line 510. Removed “, probably juveniles”.

Line 514. Changed 55 to 54 and 56 to 55.

Line 519. Changed 57 to 56.

Line 521. Changed 58 to 57.

Line 522. Changed 59 to 58.

Line 524. Changed 54 to 53.

Line 548. Changed 55 to 54.

Line 550. Changed 60 to 59.

Line 553. Changed 61 to 60.

Line 558. Changed 61 to 60.

Line 559. Changed 62 to 61.

Line 583. Changed 46 to 45.

Line 585. Changed 63 to 62, and 46 to 45.

Line 587. Changed 64 to 63.

Line 589. Change 65 to 64.

Line 591. Changed 66 to 65.

Line 593. Changed 62 to 61.

Line 620. Changed 67 to 66.

Line 621. Changed 68 to 67.

Line 622. Cahnged 51 to 50.

Line 623. Changed 69 to 68.

Line 624. Changed 70 to 69.

Line 637. Changed “side” to “sides’ ”

Line 663. Changed “articulates” to “is articulated.”

Line 686. Changed “ventral” to “the ventral.”

Line 705. Changed 71 to 70 and 72 to 71.

Lines 713 and 714. Changed “The fossil record from the Carrillo Puerto needs to be better known” to “As a result of this study, the fossil diversity from the Carrillo Puerto Formation is determined to be much greater than previously understood.”

Table 1. Added a space in “*Aetomylaeus* sp.”

Table 1. Removed an unnecessary point in “Cervidae undetermined”.

Line 752. Changed 73 to 72.

Line 754. Changed 74 to 73.

Line 756. Changed 74 to 73.

Line 758. Changed 75 to 74, and 76 to 75.

Line 759. Changed 77 to 76, and 78 to 77.

Line 760. Changed 79 to 78, and 80 to 79.

Line 761. Changed 81 to 80.

Line 762. Changed 79 to 78.

Line 767. Changed 76 to 75, and 82 to 81.

Line 768. Changed 80 to 79, and has to have.

Line 773. Changed 79 to 78.

Line 776. Changed 83 to 82.

Line 784. Changed 83 to 82.

Line 785. Changed 84 to 83, and 85 to 84.

Line 794. Changed “the adult” to “adult”.

Line 795. Changed “indicates” to “indicate”.

Line 802. Changed “speciose” to “diverse”.

Line 804. Changed 86 to 85.

Line 806. Changed 87 to 86.

Line 807. Changed 87 to 86.

Line 808. Changed 88 to 87, and 85 to 84.

Line 809. Changed 84 to 83.

Line 828. Removed the words “juvenile” and “young”.

Lines 833 to 844. Paragraph rewrite to “The teleost fish and the megalodon shark indicate the presence of small specimens in the Carrillo Puerto Formation. The upper jaw of †*Chilomycterus dzonotensis* sp. nov., for example, has only 16.5 mm in horizontal length, and the shape and number of the tooth plates in the occlusal surface indicate that the total length of the specimen should be about 10 cm, according to Tyler [15]. The maximum length of extant *Chilomycterus* species ranges from 27.9 to 69.7 cm [40]. The biggest megalodon tooth in the Carrillo Puerto Formation is about 39 mm in total length, and two specimens show a lateral cusplet only on one side.The presence of lateral cusplets was initially proposed to separate the species †*Otodus* (*Carcharocles*) *chubutensis* and †*O*. (*C*.) *angustidens* from †*O*. (*C.*) *megalodon*. Nevertheless, recent studies using life-stage tooth morphology and total length estimates of several megalodon specimens indicate this is a standard feature found in juvenile specimens of megalodon sharks [41]”.

Line 865. Reference 32 added.

Lines 865 to 867. The sentence “the co-occurrence of these species was previously reported from Miocene outcrops of North Carolina and Peruvian localities, for example, was removed.”

Line 868. References 28 and 54 added.

Lines 869 to 871. Removed the sentence, “This finding has significant implications for our understanding of the distribution and extinction of these species.”

Lines 874 and 875. Changed “wholly extinguished” to “extinct.”

Line 882. Changed 89 to 88.

Line 901. Changed the word “all” to “some.”

Line 903. Changed “a shelter, or paleo nursery, for” to “small specimens.”

Lines 925 to 929. Reference changed to “U.S. Environmental Protection Agency (USEPA) A Lexicon of Cave and Karst terminology with special reference to environmental Karst hydrology. Office of Research and Development, National Center for Environmental Assessment, Washington Office, Washington, DC, EPA/600/R-02/003. 2002. Available from https://ordspub.epa.gov/ords/eims/eimscomm.getfile?p_download_id=36359”

Lie 935. Changed to Bol Geol Min.

Lines 940 to 944. Reference changed to “López-Martínez RA, Barragán R, Rosado-González E. Sac Actun Underwater Cave System. In: The first 100 IUGS International Commission on Geoheritage, editor. International Union of Geological Sciences; 2002. pp. 260–261.”

Line 949. Changed to Rev Inst Geol.

Line 950. Changed to Servicio Geológico Mexicano (SGM)

Line 951. Removed “editors.”

Line 952. Added <https://www.sgm.gob.mx/Lexico_Es/>

Lines 953 to 957. Reference changed to Servicio Geológico Mexicano (SGM). Carta Geológico-Minera Estados de Campeche, Quintana Roo y Yucatán, escala 1:250,000 (Map). Servicio Geológico Mexicano. 2006. Available from: https://mapserver.sgm.gob.mx/Cartas_Online/geologia/peninsula_3.pdf

Line 954. Removed “; 1-39.”

Line 957. Removed “; 56p.”

Lines 962 to 966. Reference changed to “Miranda-Huerta A., Cureño-Suriano R. Carta Geológico-Minera Tizimín F16-7 Escala 1:250,000 Estado de Yucatán. Servicio Geológico Mexicano (Report). 2005. Available from: https://mapserver.sgm.gob.mx/InformesTecnicos/CartografiaWeb/T312005MIHA0001_01.PDF”

Line 964. Removed “, 84 fig., 1 tabl.”

Lines 967 to 972. Reference changed to Zarate-Barradas RG, Lemus-Bustos O, Miranda-Huerta A, Cureño-Suriano R. Carta Geológica-Minera y Geoquímica Mérida, Clave F16-10, Escala 1:250,000, Estados de Yucatán, Campeche y Quintana Roo. Servicio Geológico Mexicano (Report). 2005.Available from: <https://mapserver.sgm.gob.mx/InformesTecnicos/CartografiaWeb/T312005ZABR0001_01.PDF>.

Lines 974 and 975. Reference changed to “López-Ramos E. Estudio Geológico de la Península de Yucatán. Bol Asoc Mex Geól Petr. 1983;25: 23–76.”

Line 978. Removed the word “In”.

Line 980. Removed “, 84 fig, 1 tabl.

Lines 982 and 983. Changed to “National Oceanic and Atmospheric Administration technical Report National Marine Fisheries Service Circular 434.”

Lines 998 and 999. Removed “[sic for 113-120] + 121-124.”

Line 1000. Changed , to :.

Line 1001. Removed “;8: 549p.”

Line 1003. Removed “No.125” and added “(125).”

Line 997. Changed to “(2023): 104176.”

Line 1031. Reomved “,pls”.

Line 1046. Removed ‘pp. 1-200.”

Lines 1052. Removed the word Article.

Line 1054 to 1056. Reference changed to “Costa SAF, Richter M, Toledo PM, Moraes-Santos HM. Shark teeth from Pirabas Formation (Lower Miocene), northeastern Amazonia, Brazil. Bol Mus Par Emílio Goeldi, Ciênc Nat. 2009;4: 221–230.”

Line 1061. Added “(5):”

Lines 1066 to 1067. Removed “[ref. 3067]; 1837” and “Avaible from…”

Line 1068 to 1070. Reference changed to “Dartevelle E, Casier E. Les poissons fossiles du Bas-Congo et des régions voisines. Ann Mus Congo Belge, Sér A (Minéralogie Géologie, Paléontologie), 3. 1943;2: 1–200.”.

Lines 1071 to 1074. Reference removed.

Lines 1087 to 1090. Rerence changed to “Agassiz L. Recherches sur les poissons fossiles: une introduction a l'etude de ces animaux. Tomme I: Contenant l'introduction et toutes les questions generales, anatomiques, zoologiques et geologiques. Neuchâtel: Petitpierre; 1835.”

Line 1099. Added a space between “1964; Moscow”. Changed “Moscou” to “Moscow.”

Line 1100 to 1105. Reference changed to “52.	Agassiz L. Recherches sur les poissons fossiles: une introduction a l'etude de ces animaux. Tomme III: Contenant l’Histoire de l’Ordre de Placoides. Neuchâtel: Petitpierre; 1838.”

Line 1108. Removed “; 824 pp.”

Line 1122. Removed “; 310 pp.”

Line 1125. Changed “;” to “,”

Line 1127. Changed to “Abt. A, 168 (5–6): 149–229.”

Lines 1134 and 1135. Change to “19703(ext): 1–139. doi: 10.18563/pv.3.ext.1-139.”

Lines 1139 and 1140. Removed “pl. 18-27.”

Lines 1150 and 1151. Removed “Avaible from…”

Lines 1156 to 1158. Changed to “Prodromus systematis ichthyologiae. Nuovi Annali delle Scienze naturali Bologna. 1835;4(1): 181-196, 272-277.”

Line 1160. Removed “[ref. 296].”

Line 1173. Removed “Ed II, Stuttgart” and added “2:”.

Lines 1179 and 1180. Removed 1883 (Stuck 6, erster…”, and added “6:”.

Line 1195. Removed “Taf. 1-18.”

Line 1201. Removed “1960” and “:1-35.”

Lines 1204 to 1206. Reference changed to “Leidy J. Description of Vertebrate Remains, chiefly from the Phosphate Beds of South Carolina. Journal of the Academy of Natural Sciences of Philadelphia. 1877;2(4): 209–261.”

Line 1209. Removed “, Taf., Paris.”

Line 1211. Removed “15 Abb., Taf. 25-28, Wien.”

Lines 1212 and 1213. Changed to “Mém Suis Paleontol ” and removed “3 Pl.”
